# RNA sequencing and weighted gene co-expression network analysis uncover the hub genes controlling cold tolerance in *Helictotrichon virescens* seedlings

**DOI:** 10.3389/fpls.2022.938859

**Published:** 2022-09-02

**Authors:** Mingjun Cheng, Zeyang Pan, Kuoshu Cui, Junjun Zheng, Xuan Luo, Youjun Chen, Tao Yang, Hui Wang, Xiaofeng Li, Yang Zhou, Xiong Lei, Yingzheng Li, Ruizhen Zhang, Muhammad Zafar Iqbal, Ruyu He

**Affiliations:** ^1^Institute of Qinghai-Tibetan Plateau, Southwest Minzu University, Chengdu, China; ^2^Sichuan Grass Industry Technology Research and Promotion Center, Chengdu, China; ^3^Maize Research Institute, Sichuan Agricultural University, Chengdu, China; ^4^Sichuan Agricultural Technology Extension Station, Chengdu, China; ^5^Institute of Agricultural Information and Rural Economy, Sichuan Academy of Agricultural Sciences, Chengdu, China; ^6^Sichuan Academy of Grassland Science, Chengdu, China; ^7^College of Grassland Science and Technology, Sichuan Agricultural University, Chengdu, China

**Keywords:** *Helictotrichon virescens*, perennial herbs, cold tolerance, WGCNA, active-oxygen scavenging enzymes, homologous gene identification

## Abstract

*Helictotrichon virescens* is a perennial herbaceous plant with a life expectancy of about 10 years. It has high cold and heat resistance and can successfully survive over winter in the habitats with a temperature range of −25 to 25°C. Therefore, this study aimed to identify the key genes regulating low-temperature stress responses in *H. virescens* and analyze cold tolerant at molecular level. This study used RNA sequencing (RNA-Seq) and weighted gene co-expression network analysis (WGCNA) to identify the hub genes associated with cold tolerance in *H. virescens*. RT-PCR was conducted, homologous genes were identified, and related bioinformatics were analyzed to verify the identified hub genes. Moreover, WGCNA analysis showed that only the brown module had the highest correlation with the active-oxygen scavenging enzymes [peroxide (POD), superoxide dismutase (SOD), and catalase (CAT)]. The expression levels of three hub genes in the brown module (Cluster-37118.47362, cluster-37118.47713, and cluster-37118.66740) were significantly higher under low-temperature stress than those under control conditions. Furthermore, gene ontology (GO) and KEGG annotations showed that the three hub genes were mainly enriched in the metabolism pathways of sphingolipids, selenocompounds, glyoxylate, and dicarboxylate, carotenoids biosynthesis, and other biological pathways. The results of this study also showed that the subcellular localization prediction results showed that the cold tolerance hub genes were all localized to the plasma membrane. By constructing a protein interaction network, it was found that the hub gene Cluster-37118.66740 interacted with Sb09g003460.1 and Sb04g020180.1 proteins in *Sorghum bicolor.* By constructing phylogenetic trees of the four species of *H. virescens*, *Sorghum bicolo*, *Oryza sativa Japonica*, and *Arabidopsis thaliana*, the results showed that, the hub gene Cluster 37118.66740 (of *H. virescens*) and Os03g0340500 (of *Oryza sativa Japonica*) belonged to the same ancestral branch and were in the same subfamily. Thus, this study provides methodology and guidance to identify the cold tolerance genes for other herbage and their cold tolerant molecular mechanisms at molecular level.

## Introduction

Qinghai-Tibet Plateau region has a cold continental climate with daily temperature fluctuations. In this region, the winter is long and cold, with extremely low temperatures. Cold stress and other disasters occur from time to time, resulting in short growing period with small plants and low biomass of grassland vegetation, and even unable to survive over winter normally which leads to plant death. Low-temperature stress severely restricts plant growth and grassland renovation in Qinghai-Tibet Plateau.

*Helictotrichon virescens* is a Tetraploid perennial herb with about 100 species distributed across Asia, Europe, and North America. More than 20 species are distributed across the provinces of China; for example, there are about 11 species and one variant in Sichuan, and other nine species and one variant in the high-altitude areas of Ganzi, Aba, and Liangshan. These species are mainly distributed in the high-altitude areas of 2,000–4,500 m, where they serve as an important part of the wild pastures. *Helictotrichon virescens* has many leaves, soft blades, lower crude fiber content (than the old Mangosteen and Elysia), high nutritional value, and good palatability for ruminants. It has high economic values in terms of output and quality and has been widely promoted in the northwest plateau of Sichuan. The grass has a lifespan of about 10 years with high cold and heat resistance and can successfully overwinter in low-temperature habitats (−25 to 25°C; Cheng et al., 2022). *Helictotrichon virescens* has been playing an irreplaceable role in promoting animal husbandry in the alpine grassland, and has become an important source of high-quality forage for livestock supplement during winter and spring seasons in the alpine pastoral areas of the Qinghai-Tibet Plateau.

The process of crops adapting to low-temperature stress can be divided into several levels, such as changes in biofilm structure, cell osmotic regulators, antioxidant enzymes, and changes in photosynthesis parameters. Low-temperature stress affects the stability of the plant cell membrane systems (Hoffman et al., 2010), which is considered as the main target of low-temperature damage. The imbalance of ions and osmolates inside and outside the cell destroys the stability of the cell membrane system, which in turn affects the photosynthesis and respiration of plants. Change in the cell membrane system is the initial response mechanism of turfgrass chilling injury or freezing injury response (Levitt, 1980). Turfgrass produces the large amounts of reactive oxygen species (ROS) under low-temperature stress, including hydrogen peroxide (H_2_O_2_), superoxide radical (O^2−^), hydroxyl radical (-OH), etc., which in turn induce antioxidant enzyme systems activation, including peroxides Enzyme (POD), superoxide dismutase (SOD), catalase (CAT), and other anti-stress reactions, which collectively remove ROS and free radicals from plants, thereby alleviate the damage of turfgrass caused by low-temperature stress (Sigaud-Kutner et al., 2010). Under the stress of low temperature, plants can also reduce the cold damage by adjusting the osmotic balance of cells with the accumulation or decomposition of osmotic regulating substances. In recent years, a large number of studies have demonstrated that the contents of free proline, soluble sugar, and soluble protein are closely related to the stress tolerance mechanism in plants.

Physiological, transcriptomic, proteomic, and metabolomic changes during cold acclimation of perennial herbs have been extensively studied (Hoffman et al., 2010; Bocian et al., 2011, 2015; Paina et al., 2014; Abeynayake et al., 2015; Augustyniak et al., 2018). For example, a major quantitative trait locus QTL in *LpCBFIIIc* was associated with low-temperature tolerance, which was identified in 109 perennial ryegrass plants (Hulke et al., 2012). Candidate gene association mapping revealed that *LpCBF1b* was closely associated with winter survival, while *LpLEA3*, *LpMn SOD*, *LpCAT*, and *LpChl*, *Cu-Zn SOD* were mainly associated with spring plant regeneration (Yu et al., 2015). Heterologous overexpression of *AtDREB1A/CBF3* increased cold stress tolerance in ryegrass plants (Li et al., 2011). Transcriptomic analysis revealed that 60 cytochrome P450s transcripts were upregulated and 59 transcripts were downregulated in tall fescue under low-temperature stress, of which 26 and 17 transcripts were involved in the metabolism of flavonoids and brassinosteroids, respectively. Cold stress regulation suggests that P450s play a crucial role in the cold stress response of tall fescue (Tao et al., 2017). Zhao et al. (2020) studied cold-tolerant material “10–122” and low-temperature sensitive material “09–126” of *Poa pratensis* in native grassland of Qinghai Province by transcriptome sequencing technology to analyze differentially expressed genes between low-temperature stress and normal temperature control. The enrichment analysis of differentially expressed genes in the two materials showed that the differentially expressed genes were significantly enriched in photosynthesis, REDOX reaction, carbohydrate metabolism, cell membrane system, transporter, and sub-biological metabolism under low-temperature stress. In addition, some genes of calcium signaling regulation, hormone metabolism, signal transduction, antioxidant system, carbohydrate metabolism, and other pathways were only upregulated in cold-tolerant germplasm “10–122,” which have the potential to be used as cold-tolerant, such as *CML*, *CPK*, *CALM*, *DHAR*, *GST*, *NCED*, *SNRK2*, *BSK*, *CKX*, *BIN2*, *ARF,* and *PEK*, etc.

Due to the lack of genomic information of *H. virescens*, the current research on cold tolerance of *H. virescens* is mainly limited to physiological indicators. The molecular mechanism and regulatory network of *H. virescens* in response to low-temperature stress are still unclear. Therefore, in this study, RNA sequencing was performed on *H. virescens* after treating it under low temperature, and three hub genes significantly relating to protective enzymes were mined through the analysis strategy of WGCNA. RT-PCR validation, homologous gene mining, and related bioinformatics analysis were performed. This study lays the foundation for an in-depth understanding of the response mechanism of perennial herbs to low-temperature stress.

## Materials and methods

### Plant materials and measurements

Uniformly sized and plump *H. virescens* seeds were selected and sown into pots (21 cm in diameter and 16 cm in height; 50–60 seeds per pot). The pot mix contained a mixture of peat, pine needles, and yellow clay in a volume ratio of 3:1:1, according to Cheng et al. (2022). The seedlings with thinned to 20 plants per pot after the first leaf had fully expanded. The plants were watered daily using a half-strength Hogland nutrient solution [2.5 mmol L^−1^ Ca(NO_3_)_2_, 2.5 mmol L^−1^ KNO_3_, 1 mmol L^−1^ MgSO_4_, 0.5 mmol L^−1^ KH_2_PO_4_, 45 μmol L^−1^ Fe-EDTA, 23 μmol L^−1^ H_3_BO_3_, 4.55 μmol L^−1^ MnCl_2_, 0.16 μmol L^−1^ CuSO_4_, 0.38 μmol L^−1^ ZnSO_4_, and 0.28 μmol L^−1^ Na_2_MoO_4_]. After 5-week growth, seedlings were placed in a constant temperature and light incubator (MLR-352H-PC) simulating low-temperature stress = (0°C), with the illumination of 3000 Lx. The control temperature was 25°C. The third leaf of each plant was collected after 12, 36, and 60 h of the low-temperature treatment and immediately frozen in liquid nitrogen, and stored in a −80°C ultra-low temperature refrigerator for the determination of related indicators. Each treatment had three biological replicates.

### Phenotypic data analysis

At the end of the experiment, 30 seedlings with relatively consistent growth were selected from the treated and control groups for the relative leaf conductivity using assay developed by Cheng et al. (2022). Briefly, the middle section (2 cm × 4 cm) of the first fully expanded leaf were obtained from each seedling, and then mixed and cut into 1 cm pieces. The pieces were subsequently divided into three parts placed in 10 ml EP tubes, which were then filled with distilled water. After soaking for 3 h, the Ec1 of the leaves was measured using a conductivity meter, while the Ec2 was measured using the same instrument after the leaves being incubated in a boiling water bath for 10 min and cooled to room temperature. The relative conductivity was calculated using formula: REC = EC1/EC2 × 100%.

Chlorophyll a and b were extracted from 0.2 g of fresh leaves using 95% ethanol at room temperature, as described by Cheng et al. (2022). Briefly, the homogenate was centrifuged at 10,000 *g* for 10 min, and the chloroplast pigment extract was aliquoted into 1 cm cuvette to measure the absorbance at wavelengths 663 and 646 nm using 95% ethanol as blank. The chlorophyll concentration was calculated using formula:


$$\text{Chlorophyll a}=12.21\times {\text{OD}}_{663}-2.81\times {\text{OD}}_{646}$$

$$\text{Chlorophyll b}=20.13\times {\text{OD}}_{\text{646}}-5.03\times {\text{OD}}_{663}$$

Pro, SOD, POD, catalase (CAT), and ROS activities were measured using kits purchased from China Quanzhou Ruixin Biological Technology Co., LTD, and the type is Ruixinbio (Quanzhou, China).

The mean, standard error (SE), maximum and minimum values, and coefficient of variation, kurtosis, and skewness of each trait were calculated in Microsoft Excel. ANOVA of the collected phenotypic traits was conducted using SPSS (Statistical Product and Service Solutions, version 21.0, IBM, Armonk, NY, United States). The correlation analysis of phenotypic traits was carried out using the PerformanceAnalytics package in R software.

### RNA-seq, WGCNA, and hub gene identification

Three biological replicates of the leaf samples (third leaf of each plant collected after 12, 36, and 60 h of low-temperature treatment) stored at −80°C were processed for RNA-seq. The RNA extraction, detection, cDNA library construction, and sequencing were conducted at Beijing Nuohezhiyuan Technology Co., Ltd. Utilizing Oligo(dT) magnetic beads enriched mRNA with polyA tail procedures. Briefly, mRNA was randomly fragmented by divalent cations in NEB Fragmentation Buffer (NEB, Ipswich, MA, United States). The first strand of cDNA was prepared using random oligonucleotides as a primer, and the second strand was synthesized using DNA polymerase I. The ends of purified double-stranded cDNA were repaired by adding a tail and sequencing connector and screened to 250–300 bp cDNA for PCR amplification. The PCR product was purified again to obtain the final library, and after qualifying the library. High-quality sequencing libraries were sequenced on the Illumina HiseqTM 4,000 sequencing platform, statistical Q20 (Phred = −10log10 [e]), Q30 (Phred = −10log10 [e)], GC content and sequencing error (< 6%), and other indicators of sequencing quality control were used for these procedures (Cheng et al., 2022), and Trinity software (Grabherr et al., 2011) was used to splice clean reads into transcripts (Unigenes), which were used as reference sequences for subsequent analysis. The splicing transcript was sequenced based on its length from long to short, and the length of transcript was added to the length of splicing transcript, so that it was not <50/90% of the total length, namely N50/N90, to measure the continuity of *de novo* assembly. Its numerical value can be used to evaluate the quality of the assembly. The obtained Unigenes were functionally annotated using Blast (Altschul, 2012) on seven databases, including non-redundant (NR), nucleotide (NT), gene ontology (GO), EuKaryotic Orthologous Groups (KOG), KEGG Orthology (KO), Swiss-Prot, and FPKM. Thereafter, clean reads from each sample were aligned to the reference sequence (obtained by Trinity splicing), and the read counts of the aligned genes were obtained using Bowtie2. The number of read counts was converted to FPKM values (expected number of Fragments per Kilobase of transcript sequence per Millions base pairs sequenced; Trapnell et al., 2010) and used to evaluate the gene expression levels (FPKM > 0.3 was regarded as gene expression). Quantitative gene expression analysis was conducted using the RSEM method (Dewey and Li, 2011).

Differentially expressed genes were determined from the different samples using statistical analysis based on gene expression levels, and their original read counts were normalized with DEG-Seq software (Love et al., 2014). Negative binomial distribution (*p* value) was used for hypothesis testing, followed by multiple hypothesis test correction using Benjamini-Hochberg (BH) method to obtain the FDR value (False Discovery Rate, or padj). The obtained FDR values were then used to screen for the differential genes using |log_2_ (fold change)| > 1&padj < 0.05 as the standard.

The differentially expressed genes were constructed according to the normalized FPKM values of obtained transcriptome data from 18 cold stress treatments (including control) for weighted gene co-expression network analysis (WGCNA). The R package used for this analysis is presented in Supplementary Paper 1. The WGCNA program parameter settings were: variance data expression >0; no missing data expression <0.1; soft threshold = 10 (estimated value); maximum block size = 2,000; depth split = 4; minimum block size = 50; and Merge cut height = 0.1. Moreover, hub genes were screened in each module using the connection value (|KME|) > 0.95, module membership (MM) > 0.9, and gene significance (GS) > 0.9 of eigengenes, and functionally annotated. Cytoscape.v3.9.1 was used to draw the local transcriptional regulatory network.

### GO and KEGG enrichment hub genes

The GO enrichment analysis of the hub genes was performed using GOseq method (Young et al., 2010), while KOBAS method (Kanehisa et al., 2008) was used for the KEGG metabolism and signal transduction pathway enrichment analysis of the hub genes. In both analyses, padJ < 0.05 was used as the threshold for significant enrichment.

### Real-time fluorescence quantitative analysis of the hub genes

The RNA was extracted from the samples (stored at −80°C) using the HiPure Plant RNA Mini Kit (Magen), according to the manufacturer’s instructions. The concentration and quality (OD_260_/OD_280_ value) of the RNA samples were determined using the NanoVue plus Spectrophotometer. Subsequently, total RNA was used as a template for the first-strand cDNA synthesis using the RevertAid First Strand cDNA SynthesisKit (TaKaRa), which also contains recombinant endonuclease (DNase I) for removing genomic DNA contamination from RNA samples. Briefly, DNase I was added to the RNA samples and incubated at 37°C for 30 min for DNA removal. Thereafter, 1 μl of 50 mM EDTA was added, and the mixture was incubated at 65°C for 10 min to inactivate the DNase I. Reverse transcription was then performed by adding the reverse transcriptase to the mixture and incubating at 42°C for 60 min. The samples were then stored at −20°C for later use. The reverse transcription process is shown in Supplementary Paper 2.

The real-time fluorescence quantitative (qRT-PCR) reaction system (CFX96 real-time PCR system; Bio-Rad) was sterilized before configuration, and the samples were mixed and loaded into the instrument. The real-time fluorescence quantitative reaction system is shown in Supplementary Paper 3. The reaction involved three biological and four technical replicates, as shown in the procedure presented in Supplementary Paper 4. The qRT-PCR results were analyzed by the 2^-△△Ct^ method with GAPDH as the internal reference; it is widely present in many organisms and is abundant in cells, accounting for 10–20% of the total protein. The GAPDH gene has a highly conserved sequence, and the protein expression level in the same cell or tissue is generally constant. Therefore, this gene has been widely used as an internal reference gene in qPCR for a long time. In this study, GAPDH is the internal reference gene of naked oats (from the Wheat Research Institute of Sichuan Agricultural University).

### Homologous EST label identification of hub gene

The Open Reading Frame Finder tool from NCBI was used to align the RNA-Seq data to the open reading frames (ORFs) of the transcripts obtained from the reference genomes of *Avena barbata* and *Hordeum vulgare* subsp. The functions of the expressed genes are determined by comparing and analyzing the obtained Expressed Sequence Tag (EST) with the known sequences in various public databases. The ORFs corresponding to the previously screened hub gene were used to search the EST and Genbank databases on the NCBI official website^1^ to obtain the hub genes. Thereafter, the matched base sequences were screened for homologous genes, using the alignment thresholds of identity>85% and Query Coverage>30%.

### Bioinformatics analysis of hub genes and construction of protein interaction network

First, extract the hub genes sequence from the non-redundant gene database obtained by RNA-seq sequencing and splicing of *H. virescens*. Then, the hub genes sequence was aligned to the NR database and the Swissprot protein database, the ORF coding frame information corresponding to the transcript was extracted from the alignment result, and the coding region sequence was translated into the amino acid sequence according to the standard codon table (according to the 5′- >3′ order). Finally, the ORF of the hub gene was predicted by ESTSCAN^2^ software based on PSM and codon preference, there by predicting the nucleic acid sequence and amino acid sequence encoded by the hub genes.

The online analysis website ExPASy^3^ was used to predict the protein physicochemical properties of the hub gene; Subcellular localization prediction was performed using WoLF PSORT^4^; Protein transmembrane domain prediction was performed by the online tool TMHMM 2.0^5^; The online analysis website STRING^6^ was used, the monocot genome annotation information was selected as the background file for comparison, and the protein interaction network was constructed for the hub genes.

### Identification and phylogenetic analysis of homologous genes of hub genes

The predicted amino acid sequence of the hub genes was used as a probe, the *Sorghum bicolor*, *Oryza sativa Japonica*, and *Arabidopsis thaliana* were identified by alignment and domain alignment in pfam, CDD and SMART databases, and homologous genes were located with similarity >50 and E-value ≤ 1e−10. Among them, data files were downloaded from the National Center for Biotechnology Information (National Center for Biotechnology Information) database. After multiple sequence alignment using mafft software, the phylogenetic tree of the gene family was constructed by JTT + G4 method using IQ-tree software, and the phylogenetic tree was beautified using EvolView online website platform.

## Results

### Evaluation of cold-tolerance phenotypes of *Helictotrichon virescens*

**Figure 1:**
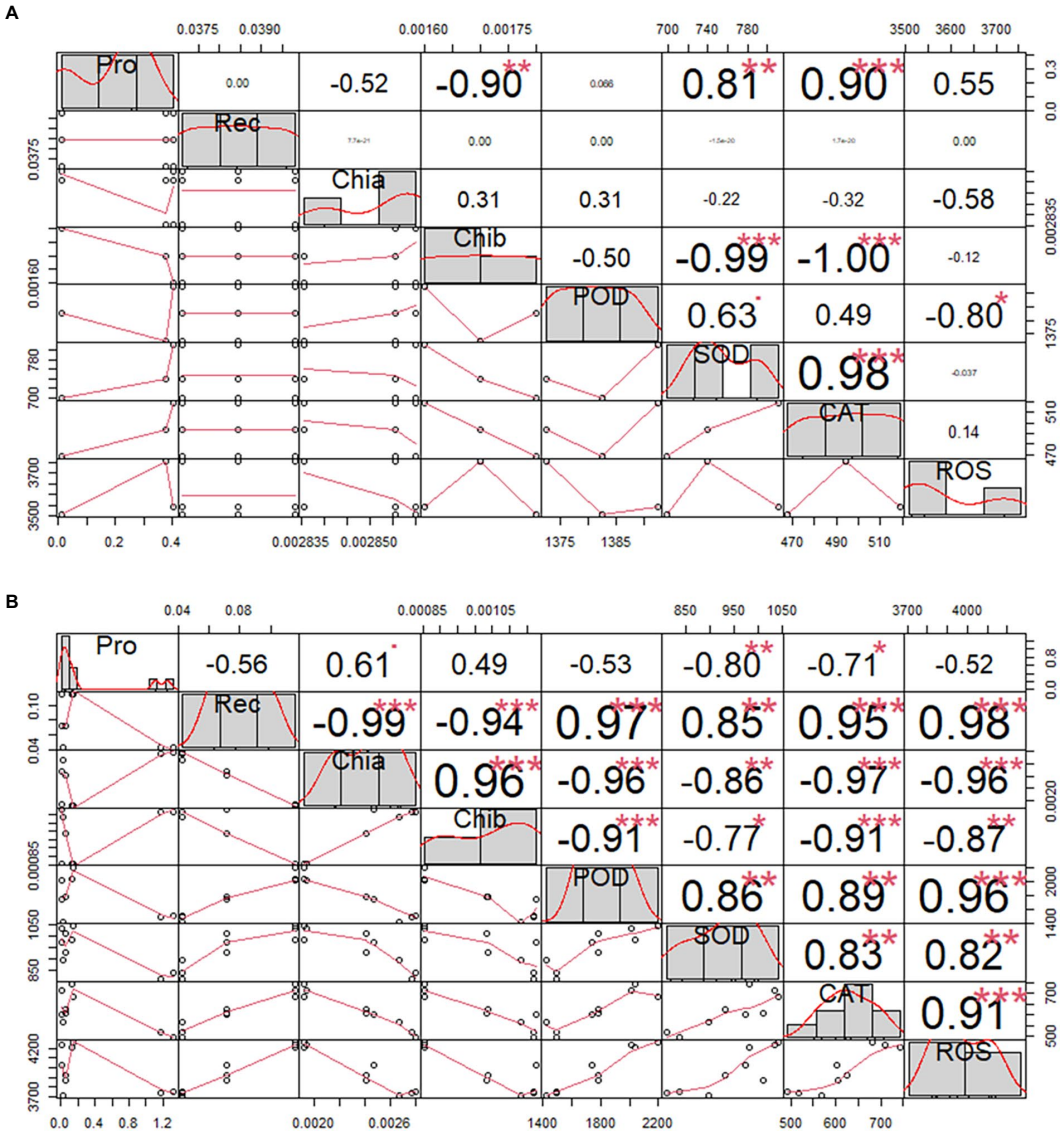
Correlation analysis of *Helictotrichon virescens* phenotypic indicators. Morphological data obtained from samples under **(A)** the control temperature (25°C) and **(B)** low-temperature stress (0°C). * represents *p*<0.05,** represents *p*<0.01 and *** represents *p*<0.001.

The cold tolerance of *H. virescens* was evaluated at its seedling stage in a simulated cold-stress environment in the laboratory. The results showed that there were no significant differences in Pro, Rec, Chla, Chlb, POD, SOD, CAT, and ROS levels in *H. virescens* seedlings grown at 25°C (Table 1). The coefficient of variation of Pro was the largest (0.2397), while POD was the smallest (0.0021); however, the kurtosis of each indicator was similar. Moreover, ROS exhibited the largest skewness (0.7980), and Chlb and POD had the smallest (0.0000). As shown in Figure 1A, Pro had a significant negative correlation with Chlb (*p* < 0.001), but positively correlated with SOD (*p* < 0.001) and CAT (*p* < 0.0001). Meanwhile, Chlb significantly negatively correlated with SOD (*p* < 0.001) and CAT (*p* < 0.001). POD and SOD showed a significant positive correlation (*p* < 0.05), while SOD and CAT showed a highly significant positive correlation (*p* < 0.001). There was no correlation between the other indicators.

**Table 1:** Statistical description of *Helictotrichon virescens* phenotypic data at 25°C.

Trait	Range	Mean	SE	CV/%	Kurtosis	Skewness	Sig
Pro	0.0115–0.4040	0.2635	0.0631	0.2397	−1.7140	−0.8400	ns
Rec	0.0371–0.0398	0.0385	0.0004	0.0100	−1.7140	0.0130	ns
Chla	0.0028–0.0029	0.0029	0.0000	0.0013	−1.7140	−0.7480	ns
Chlb	0.0016–0.0018	0.0017	0.0000	0.0170	−1.7140	0.0000	ns
POD	1372.48–1392.48	1382.4800	2.8868	0.0021	−1.7140	0.0000	ns
SOD	698.47–811.64	749.7333	16.5500	0.0221	−1.7140	0.3990	ns
CAT	467.93–518.90	493.6233	7.3576	0.0149	−1.7140	−0.0360	ns
ROS	3507.94–3755.02	3601.4067	38.7033	0.0107	−1.7140	0.7980	ns

ns, not significant.

The phenotypic data obtained after 12, 36, and 60 h under low-temperature stress (0°C) are shown in Table 2. Rec, Chia, Chib, POD, CAT, and ROS showed significant differences in treatment time gradient (*p* < 0.01), except for Pro and SOD levels. In addition to, pro had the largest coefficient of variation between the treatment time gradients, while ROS exhibited the smallest. Moreover, the kurtosis was the largest (0.8250) for Pro, but the smallest (−1.8650) for ChIb. Pro also had the largest skewness (1.6090), while SOD had the smallest (−0.5600). Furthermore, there was no correlation between Pro and POD, and Pro and ROS, except for Pro and Rec, as shown in Figure 1B.

**Table 2:** Statistical description of *H. virescens* phenotypic data at 0°C.

Trait	Range	Mean	SE	CV/%	Kurtosis	Skewness	Sig
Pro	0.0056–1.3260	0.3233	0.1763	0.5451	0.8250	1.6090	ns
Rec	0.0426–0.1155	0.0765	0.0106	0.1386	−1.7140	0.3080	**
Chla	0.0019–0.0028	0.0024	0.0001	0.0510	−1.6560	−0.3000	**
Chlb	0.0009–0.0012	0.0010	0.0000	0.0459	−1.8650	−0.3990	**
POD	1423.07–2195.00	1774.6511	89.9860	0.0507	−1.2050	0.1680	**
SOD	811.64–1043.80	947.4278	27.8088	0.0294	−1.0020	−0.5600	ns
CAT	494.04–743.17	616.8478	28.0549	0.0455	−0.9540	0.0610	**
ROS	3707.94–4269.36	3972.1533	75.2806	0.0190	−1.8280	0.2500	**

ns, not significant.

** *p* < 0.01.

Table 3 shows that Pro, Chia, Chib, POD, SOD, CAT, and ROS were significantly different between the treated and control groups (*p* < 0.01), the results of the above studies indicated that *H. virescens* had less low temperature damage and stronger resistance.

**Table 3:** ANOVA of *H. virescens* phenotypic data (Treatment vs. Control).

	SS	df	MS	F	Sig
Pro	1.2350229	5	0.2470	2.2707	0.0000
Rec	0.0146118	5	0.0029	-	-
Chla	2.098E-06	5	0.0000	386.4508	0.0000
Chlb	2.157E-06	5	0.0000	69.7016	0.0000
POD	1250555.2	5	250111.0380	119.3142	0.0000
SOD	222368.21	5	44473.6421	18.4626	0.0000
CAT	119992.54	5	23998.5080	32.3438	0.0000
ROS	1009380.7	5	201876.1399	19.3729	0.0000

### RNA-seq, WGCNA, and hub gene identification

High-throughput illumina sequencing of 18 *H. virescens* samples generated 24,000,000 bp of raw data, from which 22,730,000 bp of high-quality sequence data (clean data) were obtained after assembly and de-redundancy. The clean reads were over 6.5 G per sample, and the base error rate of the sequences from each sample was 0.02–0.03%. Additionally, the Q20 and Q30 of the clean reads were over 98.00 and 94.00%, respectively, and their GC contents were 50.21–55.21%. Trinity splicing generated 396,649 transcript sequences from the clean reads, which were used as reference sequences for subsequent analyses. These transcript sequences were assembled into 112,775 Unigenes after Corset hierarchical clustering (Cheng et al., 2022). The alignment efficiency of the clean reads from the 18 *H. virescens* samples with the reference genome was high (>70%).

**Figure 2:**
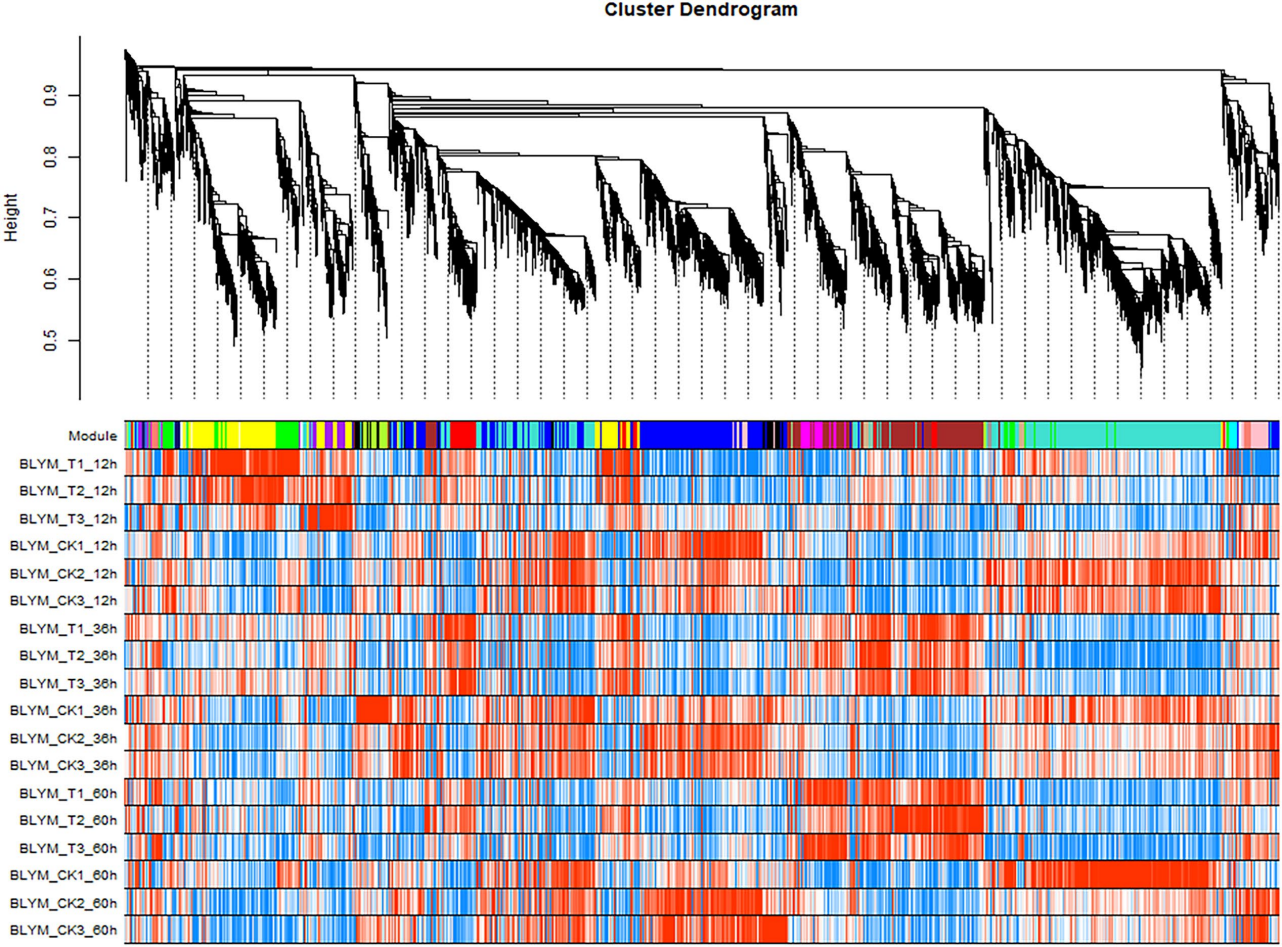
*Helictotrichon virescens* transcript clustering and module identification. The different colors represent different modules, whereby the grey module denotes a collection of differential genes not assigned to other modules. The turquoise module gathered the most genes (2,714 differential genes), while orange, dark-orange, dark-grey, and dark-turquoise modules gathered the least genes (13 genes), among which each module gathered 366 genes on average.

The expression modules were divided according to the standard of mixed dynamic shearing, and 38,921 transcripts exhibiting high expression levels were selected for subsequent analyses. The results showed that 27 gene co-expression modules were identified in this study (Figure 2).

**Figure 3:**
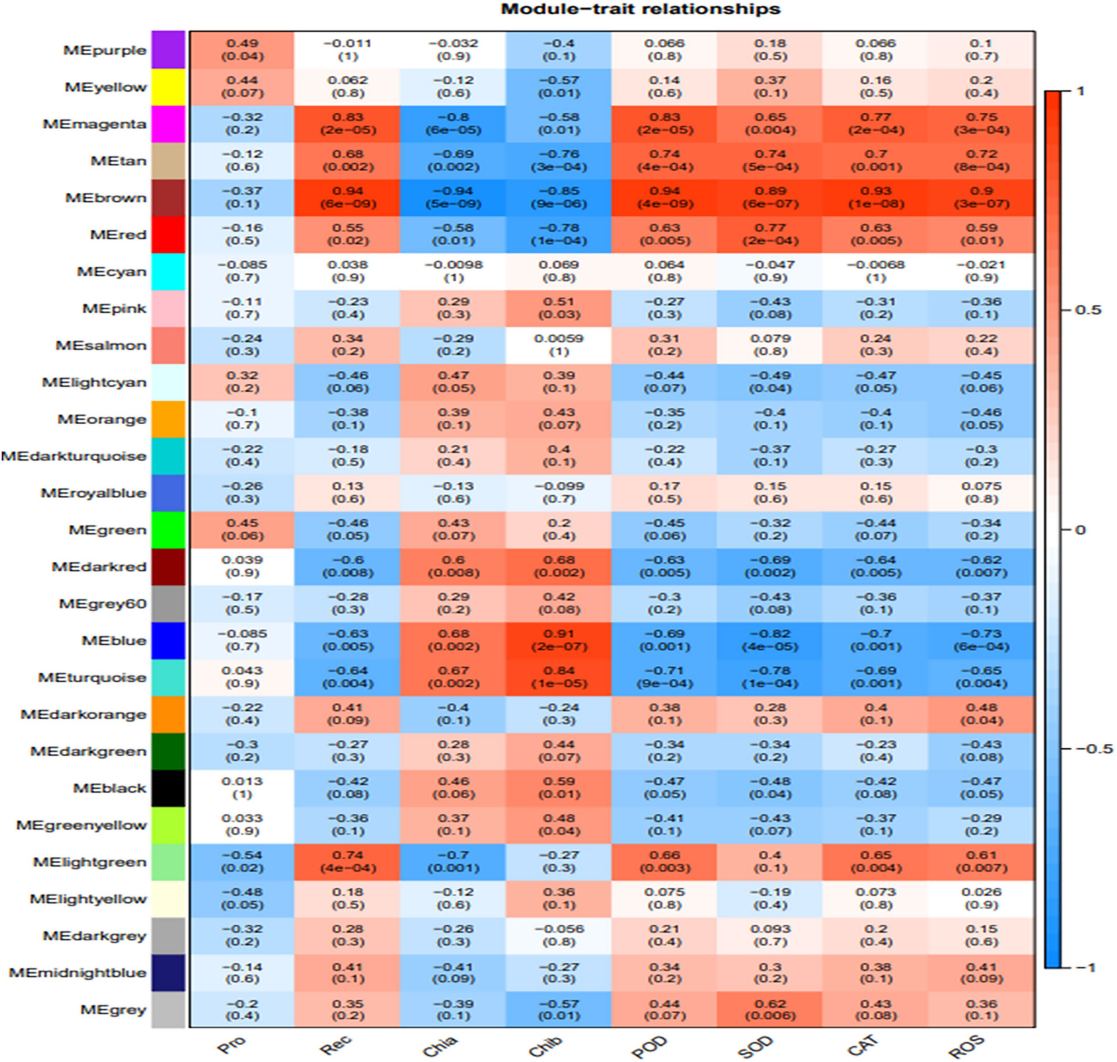
Association modules of *Helictotrichon virescens* phenotypic modules. Each row represents a module, while each column represents a trait. The numbers in the rectangular boxes represent the correlation coefficients and corresponding *p* values between the modules and traits.

The association analysis was conducted using the phenotypic traits based on the previously divided modules. The results showed that the brown, magenta, and tan modules had significant positive correlations (*p* < 0.01) with ROS and the main ROS scavenging enzymes, such as SOD, POD, and CAT. Among these, the brown module had the highest correlation with ROS and the main ROS scavenging enzymes, which were 0.94 (*p* = 4e−9), 0.89 (*p* = 6e−7), 0.93 (*p* = 1e−08), and 0.9 (*p* = 3e−07) for SOD, POD, CAT, and ROS, respectively. Furthermore, the turquoise and blue modules exhibited significant positive correlations with the chlorophyll a (Chla) and b (Chlb) contents, and the relative electrical conductivity (Rec) of *H. virescens* leaves. The other modules negatively correlated with the measured plant parameter (Figure 3). Therefore, this study selected three modules (turquoise, blue and brown modules) that exhibited the highest correlation with cold tolerance phenotype for further analysis. Among them, the brown module had 108 hub genes, while the blue and turquoise modules had 96 and 21 hub genes, respectively.

### GO enrichment hub genes

**Figure 4:**
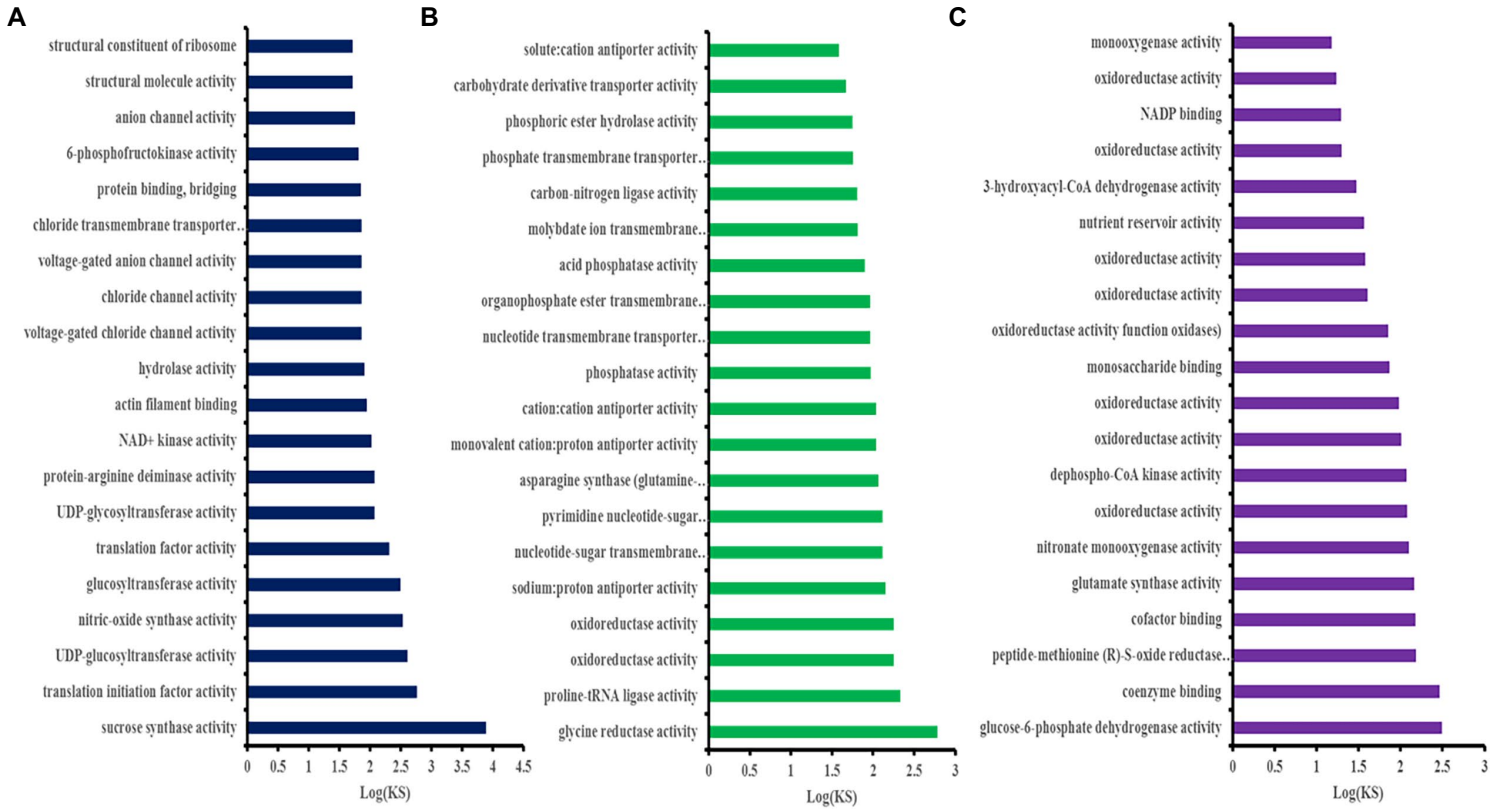
Gene ontology (GO) enrichment analysis of the *Helictotrichon virescens* hub genes (MF of the top 20 clusters) from the brown **(A)**, blue **(B)**, and turquoise **(C)** modules.

The GO enrichment analysis was performed on the hub genes obtained in the brown, blue, and turqouise modules. As shown in Figure 4A, the 108 hub genes of the brown module were mainly enriched in sucrose synthase, translation initiation factor, and biological pathways activities, such as UDP-glucosyltransferase, nitric-oxide synthase, glucosyltransferase, translation factor, structural molecule activities, and structural constituent of ribosome. Conversely, the 96 hub genes of the blue module were mainly enriched in glycine reductase, proline-tRNA ligase, oxidoreductase, and other activities of the biological pathways, such as sodium: proton antiporter, phosphatase, and carbohydrate derivative transporter activities (Figure 4B). Figure 4C shows that the GO enrichment analysis of the 21 hub genes of the turquoise module was significant in glucose-6-phosphate dehydrogenase, peptide-methionine (R)-S-oxide reductase, glutamate synthase, nitronate monooxygenase, and oxidoreductase, and other activities of biological pathways.

**Figure 5:**
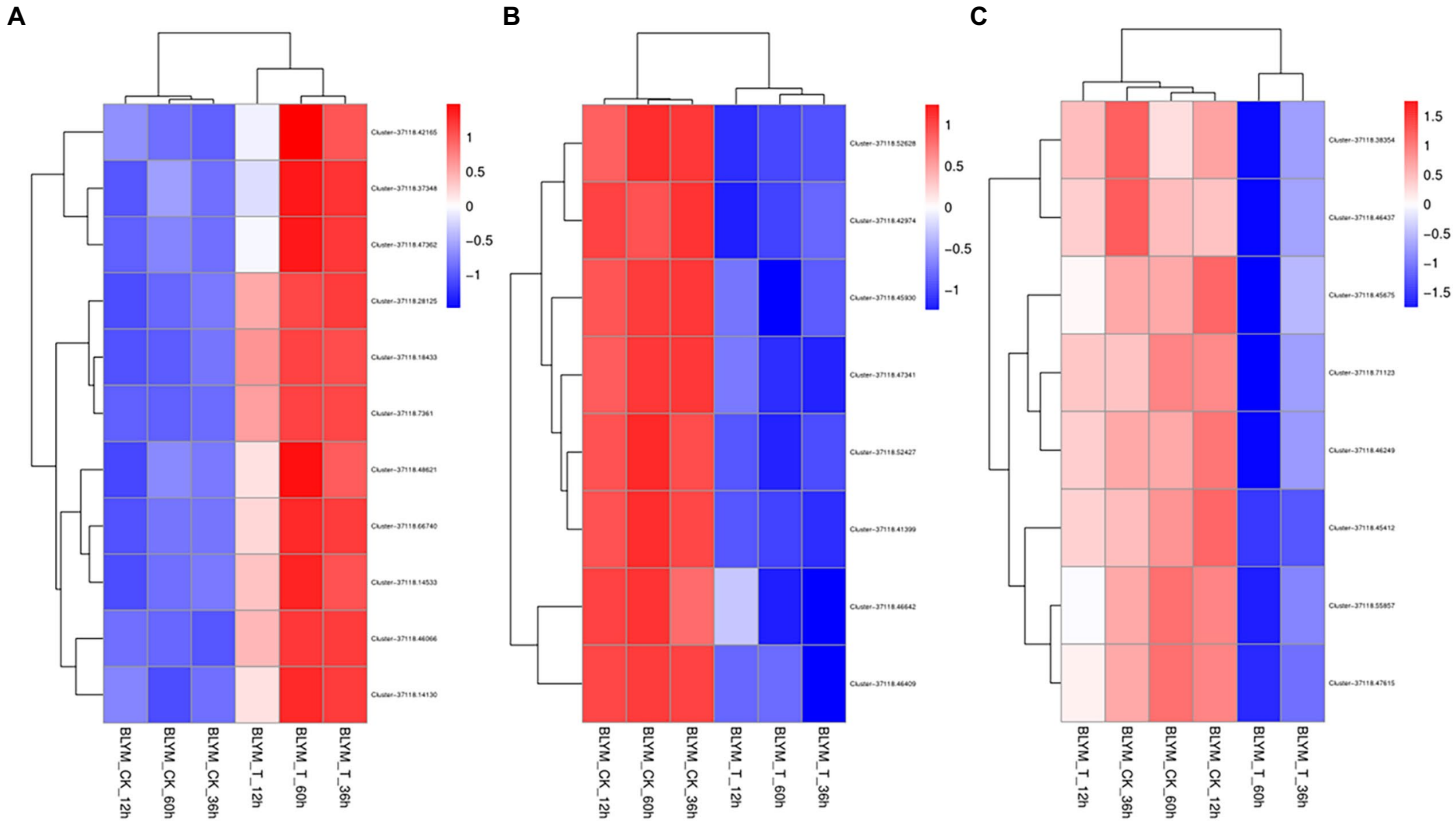
Cluster analysis of the *Helictotrichon virescens* hub genes from the brown **(A)**, blue **(B)**, and turquoise **(C)** modules.

As shown in Supplementary Table 1, clusters 37118.66740, 37118.7361, 37118.42165, 37118.46066, 37118.37348, 37118.47362, −37118.48621, 37118.14130, 37118.46066, 37118.14533, and 37118.28125 of the brown module were significantly enriched in sucrose synthase, Translation Initiation Factor, UDP-Glucosyltransferase, UDP-glycosyltransferase, and other activities of the biological pathways. The expression levels of the above 11 hub gene clusters were significantly higher under low-temperature stress than those under normal temperature (Figure 5A), indicating the important roles of these hub genes in the coercion process.

Clusters −7118.46642, 37118.47341, 37118.45930, 37118.46409, 37118.52628, 37118.52427, 37118.42974, and 37118.41399 of the blue module were significantly enriched in glycine reductase, proline-TrNA ligase, oxidoreductase, sodium: proton antiporter, and other activities of the biological pathways (Supplementary Table 2). As shown in Figure 5B, the expression levels of these hub gene clusters were significantly lower under the low-temperature treatment than those under control conditions.

As shown in Supplementary Table 3, the turquoise module had eight clusters (37118.55857, 37118.71123, 37118.46249, 37118.38354, 37118.47615, 37118.46437, 37118.55857, 37118.71123, 37118.46249, 37118.38354, 37118.47615, 37118.46437, 37118.45675, and 37118.45412), which were significantly enriched in army-6-phosphate dehydrogenase and glutamate synthase, nitronate monooxygenase, xidoreductase, and other activities of the biological pathways. The expression levels of these hub gene clusters were significantly lower under the low-temperature stress than those under control conditions (Figure 5C).

Cheng et al. (2022) showed that *H. virescens* has a very high cold tolerance and can successively survive a cold environment of up to −25°C. The 11 hub genes of the brown module were the only ones with significantly higher expression under low-temperature stress, while those from the other two modules had lower expression levels under low-temperature stress than those under control conditions. Therefore, the 11 hub genes of the brown module could be used as key genes responsible for cold tolerance in *H. virescens*, and might need to be studied further.

### KEGG enrichment hub genes

**Figure 6:**
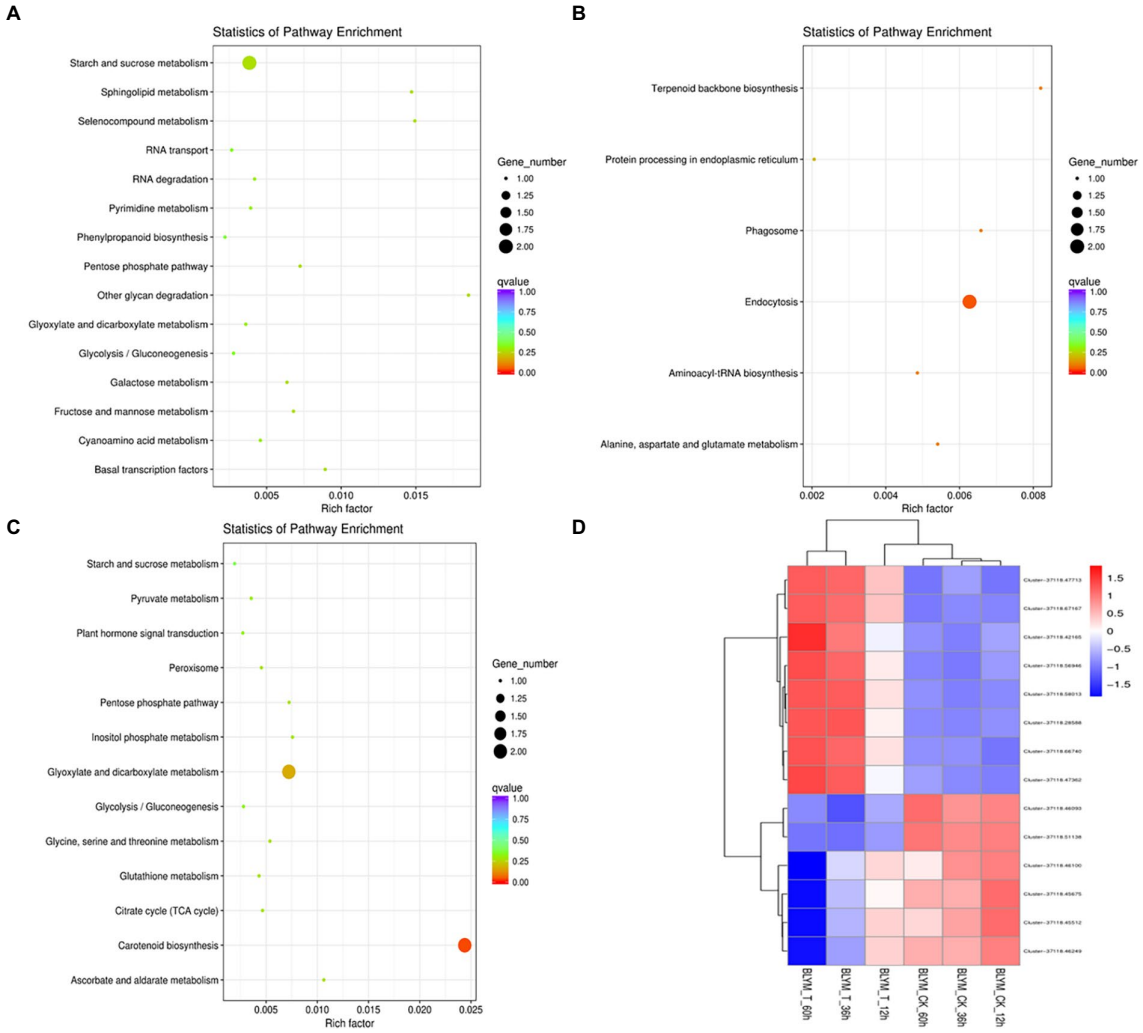
KEGG enrichment analysis and clustering analysis of *Helictotrichon virescens* hub genes from the brown **(A)**, blue **(B)** represents, and turquoise modules **(C)**. Cluster analysis representing the various hub genes **(D)**.

The KEGG enrichment analysis was performed for the hub genes in the brown, blue, and turquoise modules. As shown in Figure 6A and Supplementary Table 4, the eight hub genes in the brown module were mainly enriched in sphingolipid, selenocompound, sphingolipid, starch, sucrose, pentose phosphate, fructose, mannose, and galactose metabolism pathways, and other biological pathways. Notably, these biological pathways are all related to glucose metabolism, showing that monosaccharide or polysaccharide metabolism is involved in *H. virescens* response to low-temperature stress.

Contrarily, two out of the 96 hub genes in the blue module were mainly enriched in the endocytosis pathway (Figure 6B and Supplementary Table 5), while four out of the 21 hub genes in the turquoise module were mainly enriched in the carotenoid biosynthesis, and glyoxylate and dicarboxylate metabolism pathways (Figure 6C and Supplementary Table 6).

As shown in Figure 6D, the 14 hub genes enriched by KEGG analysis from the three modules were further subjected to cluster analysis based on their expression levels. The expression levels of clusters 37118.66740 and s37118.47362 were significantly higher under the low-temperature treatment than those under control conditions. However, the expression levels of the remaining hub genes, of which eight belonged to the brown module, were significantly lower under the low-temperature treatment than those under control conditions. Figure 3 shows that the brown module has the highest correlation with the main ROS scavenging enzymes, indicating that these enzymes play important roles in *H. virescens* response to low-temperature stress.

### Real-time fluorescence quantitative analysis of hub gene

**Figure 7:**
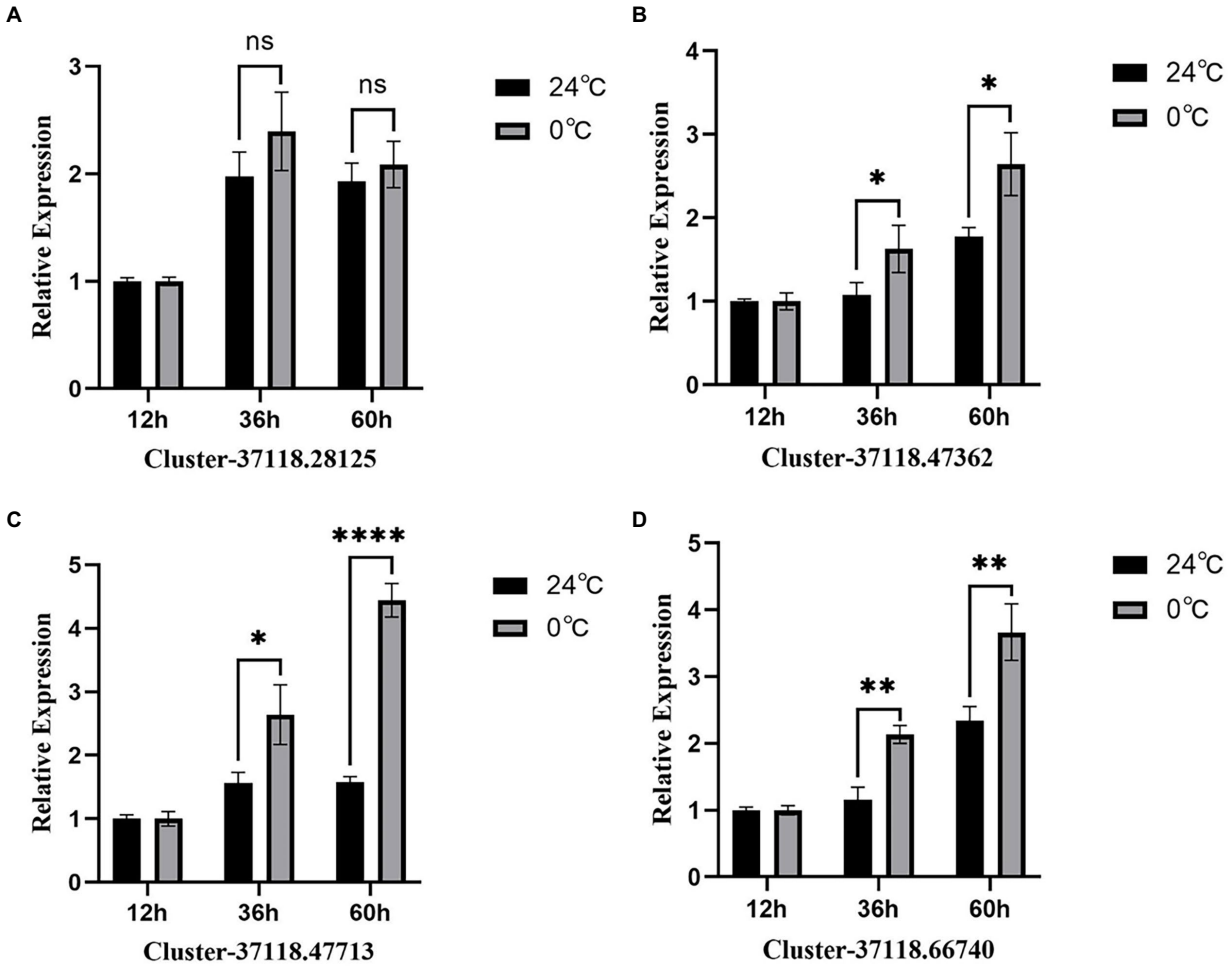
RT-PCR analysis of the four hub genes from the brown module. **(A–D)** are the expression results of four hub genes, respectively. * represents *p*<0.05,** represents *p*<0.01,*** represents p<0.001, **** represents *p*<0.0001.

In this study, the RT-PCR analysis of the four hub genes, which exhibited higher expression in the brown module (Figure 7), showed that, the expression of GAPDH was stable at different time points. However, after 12 h of low-temperature treatment, clusters 37118.47362, 37118.47713, and 37118.66740 had no significant difference between the control and treated groups. However, after 36 and 60 h of low-temperature treatment, the clusters exhibited significant differences between the control and treated groups.

### Local regulatory network of the hub genes

**Figure 8:**
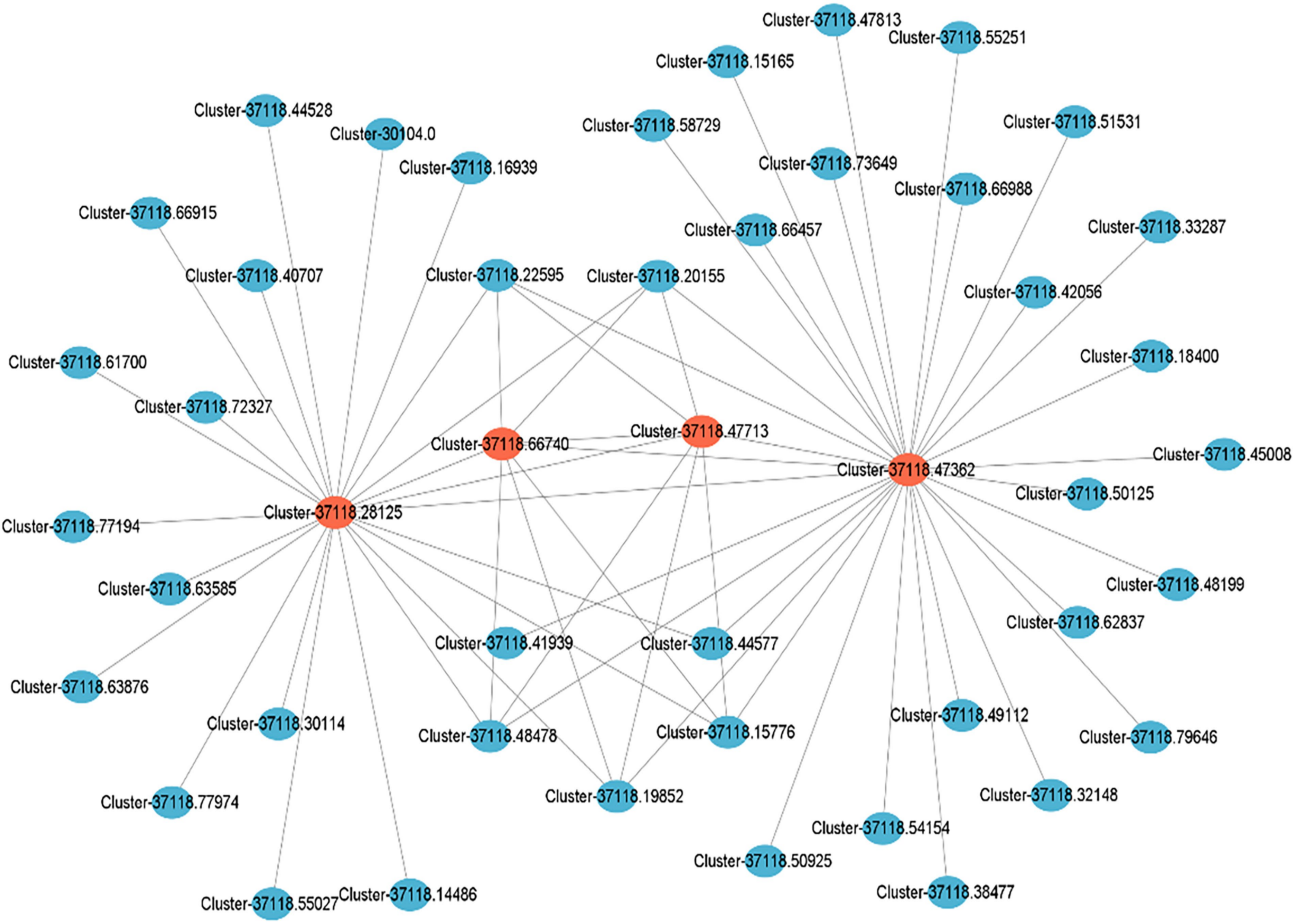
Local regulatory network of cold tolerance-related genes obtained from the brown module. Red circles represent the key cold tolerance-related genes, while blue ones represent the differentially expressed genes interacting with the key genes.

Cytoscape_v3.9.1 was used to draw a local regulatory network map of cold tolerance-related pathways based on the four key regulatory genes obtained by GO and KEGG enrichment analyses from the brown module (Figure 8). The results showed that clusters 37118.28125 and 37118.47362 are related to cold tolerance and had higher weights and more interactions with multiple genes.

### Homologous EST label identification of the hub genes

The comparison analysis of the generated hub gene transcripts shows that the four hub genes are closely related to *Triticum aestivum*, *Hordeum vulgare* subsp. *vulgare*, and the ESTs of *Avena barbata*. Gene Cluster-37118.28125 matched 21 homologous ESTs, among which it had a higher homology with cDNA sequence DK620133.1 obtained from the shoot tips of low-temperature barley subspecies seedlings. Meanwhile, gene cluster-37118.66740 matched 52 homologous ESTs, and had higher homology with DK600269.1 sequence from the cDNA library of the stem segments of low-temperature barley subspecies. The cDNA sequences obtained from callus cloning of the low-temperature barley subspecies also had high homology with gene cluster-37118.66740. The gene cluster-37118.47713 identified five homologous ESTs from oat, while gene cluster-37118.47362 matched 92 homologous ESTs, of which 11 were homologous sequences related to abiotic stress. The ESTs were deposited in the GenBank database (NCBI), and their sequences are shown in Supplementary Table 7.

### Bioinformatics analysis of hub genes

The physicochemical properties of the protein encoded by the hub genes were analyzed. The results showed that the number of amino acids in Cluster-37118.47362 was relatively small, and the number of amino acids in Cluster-37118.66740 was relatively large. In addition, the protein isoelectric points of the four hubs were all greater than 5, and they were all hydrophobic proteins (Table 4). The subcellular localization prediction results showed that, the hub genes were all localized to the plasma membrane.

**Table 4:** Physical and chemical properties and subcellular localization prediction of hub gene protein related to cold tolerance in *H. virescens.*

Transcript ID	Amino acid amounts(aa)	Molecular weight (KD)	pl	Hydrophilicity coefficient	Transmembrane domain amounts	Subcellulat localization
Cluster-37118.28125	372	42.06	6.4	−0.381	3	Plasma membrane
Cluster-37118.47362	80	39	9.89	−0.035	1	Chloroplast
Cluster-37118.66740	744	36.65	5.93	−0.243	2	Plasma membrane
Cluster-37118.47713	306	27.61	9.06	−0.412	1	Mitochondrion

### Construction of protein interaction network of hub genes

**Figure 9:**
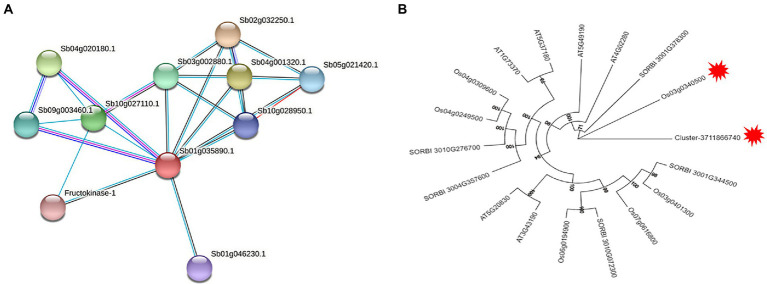
**(A)** Sb01g035890.1 protein interaction network. The red node in the figure is the sorghum protein Sb01g035890.1 aligned by Cluster-37118.66740, the red line represents gene fusion, the cyan line is from the database, the pink line is the experimental proof result, the black line is the co-expressed protein, and the blue line represents the gene symbiotic relationship; **(B)** Phylogenetic Analysis of sucrose synthase gene family.

According to the protein function annotation information of the *Sorghum bicolor* genome by STRING software, it was found that Cluster-37118.28125, Cluster-37118.47362, and Cluster-37118.47713 could not be compared to monocotyledonous crops. However, the predicted results of Cluster-37118.66740 protein were relatively accurate and the protein was highly similar to Sb01g035890.1, a key regulatory protein in the sucrose metabolism pathway in *Sorghum bicolor*. By constructing a protein interaction network, it was found that the hub gene Cluster-37118.66740 interacted with Sb09g003460.1 and Sb04g020180.1 proteins in *Sorghum bicolor* (Figure 9A).

### Identification and phylogenetic analysis of homologous genes of hub genes

A total of five sucrose synthase activity (SUS) family members were identified in *Sorghum bicolor*, 6 in *Oryza sativa Japonica*, and six *in Arabidopsis thaliana* using the protein sequence of the cold hub gene Cluster-37118.66740 as a probe (Supplementary Table 8). By constructing phylogenetic trees of the four species of *H. virescens*, *Sorghum bicolo*, *Oryza sativa Japonica*, and *Arabidopsis thaliana*, the results showed that, the hub gene Cluster 37118.66740 (of *H. virescens*) and Os03g0340500 (of *Oryza sativa Japonica*) belonged to the same ancestral branch and were in the same subfamily (Figure 9B). In addition, monocotyledonous and dicotyledonous plants form different branches, and *Oryza sativa Japonica*, *Sorghum bicolo* and *H. virescens* are more closely related. The results of this study also showed that the clustering distribution of species in the subfamily was obvious, indicating that the differentiation time of the sucrose synthase family genes was earlier.

## Discussion

### Relationship between physiological indices and response to low-temperature of *Helictotrichon virescens*

Abiotic stresses affect plant growth and development at different levels. Excessive production and accumulation of ROS (such as OH^−^, H_2_O_2_, and O^2−^) in plant cells leads to the damage of macromolecules, including proteins, RNA, and DNA. Superoxide radicals (O^2−^) are disproportionated by SOD to H_2_O_2_ and further scavenged by CAT and peroxides such as POD by conversion to H_2_O. Freezing treatment for 3 h significantly increased the activities of CAT, POD, and SOD. These results are consistent with the findings of Bermudagrass, which showed that treatment at 4°C over6 days increased the activities of CAT, POD, and SOD (Cheng et al., 2016). However, 4°C over 72 h low-temperature treatment increased SOD activity but decreased CAT and POD activities (Wei et al., 2015). These differences may be attributed to detailed changes in temperature, treatment time, and grass species. All data suggest that low-temperature stress modulates ROS by modulating antioxidant enzyme activities. Cheng et al. (2022) showed that with the prolongation of stress time, the ROS content in *H. virescens* leaves gradually increased, and the difference between the treatment and control groups reached significant or highly significant levels; at the same time, POD, SOD, and CAT were significantly enhanced, which is conducive to the scavenging of ROS in the *H. virescens* plants and other plants. In addition, due to the diversity of plant cold tolerance indicators and physiological adaptation mechanisms, the analysis of a single index often cannot truly reflect the cold tolerance of plants. Therefore, a comprehensive analysis of the related indicators of its cold tolerance can comprehensively evaluate and compare the cold resistance of plants. However, there is no report on the comprehensive evaluation of the physiological response and cold tolerance of forage varieties to low-temperature stress. He et al. (2021) used the membership function method to calculate the fresh weight, dry weight, chlorophyll content, SOD activity, MDA content, free proline content, and soluble sugar content of six kinds of grasses (*Elymus sibiricus’Qingmu No.1′*, *E.sibiricus’Tongde’, E.breviaristatus’Tongde’*, *Poa crymophila’Qinghai’*, *P.pratensis* var.*anceps Gaud’Qinghai’*, and *Festuca sinensis’Qinghai’*) under low-temperature stress. According to the membership function value of the six species of grasses, the cold resistance of the six types of grass can be comprehensively evaluated so that the evaluation results can more comprehensively reflect the actual cold resistance of the six grass species. Cheng et al. (2022) showed that the chlorophyll content in *H. virescens* leaves showed a decreasing trend with the extension of the stress time. After 12 h of low temperature stress, the content of chlorophyll a was not significantly different between the treated group and the control group, and the content of chlorophyll b was significantly reduced; after 36 and 60 h of low temperature stress, the content of chlorophyll a and b in the treated group were significantly lower than those in the control group; In addition, the relative conductivity of leaves increased after low temperature stress. After 12 h of low temperature stress, the relative conductivity of leaves in the treated group was 1.15 times that of the control group. After prolonged low temperature stress to 60 h, the relative conductivity of leaves in the treated group increased. to 2.90 times that of the control group, indicating that the stability and tolerance of leaf cell membranes were damaged during low temperature stress; Under low temperature stress, the accumulation of proline (Pro) can reduce the cytosolic freezing point and increase the osmotic potential, thereby stabilizing the cell membrane system to prevent cell freezing and dehydration and reduce the exudation of solutes. The Pro content in the treated group and the control group showed a very significant difference, and the Pro content in the treatment group was 2.16 times that of the control group. In this study, as shown in Table 3, the method analysis of the phenotypic indicators of *H. virescens* under low-temperature stress and control treatments was carried out, and the results showed that Pro, Chia, Chib, POD, SOD, CAT, and ROS were significantly different between the treated and the control groups (*p* < 0.01), the results of the above studies indicated that *H. virescens* had less low temperature damage and stronger resistance. As shown in Figure 1B, except for Pro and Rec, there was no correlation between Pro and POD, and Pro and ROS, and there were significant (*p* < 0.05) andextremely significant (*p* < 0.01) between other indicators. The above results indicate that these indicators can be used as phenotypic indicators for the identification of cold stress in *H. virescens*.

### WGCNA is a key strategy to excavate cold tolerance genes in *Helictotrichon virescens*

Weighted Gene Co-expression Network Analysis is a research method in systems biology with many applications, including mining gene modules related to target traits in multi-sample transcriptome data. Unlike other network analysis methods, WGCNA can specifically identify genes associated with the target traits, screen core genes, and perform modular classification to obtain co-expression modules with high biological significance. Therefore, choosing a quantifiable target trait is a primary consideration for WGCNA analysis.

Qin et al. (2020) used the seedlings of cultivars C16 (CIP 397077.16) and C119 (CIP 398098.119) from the International Potato Center as test materials for the mannitol-induced drought stress experiment. The study obtained 15 gene co-expression modules closely related to root drought resistance, and several core genes with the highest correlation with the target trait were identified from four modules. The functional annotation showed that most of these genes were involved in the drought stress regulation pathway. Moreover, Li et al. (2019) used 47 transcriptome data sets of normal rice tissue for cold, drought, and salt stress treatment. The study identified 15 modules using WGCNA and found that the three known rice-related genes were present in each module. Two modules related to the three stress treatments were selected to construct the gene regulatory network in which 25 key genes related to stress resistance were predicted. Wang et al. (2021) used different peanut varieties as materials to analyze the similarities and differences in the expression of the stem-growth-related genes. The results showed that the dwarf type Df216 had 5,872 differential genes while tall type Huayu had 33. Df216 and the intermediate type Shanhua 108 had 6,662 differential genes, which were implicated in the biological origin and regulatory processes of the primary and secondary cell walls biosynthesis, phenylpropane biosynthesis and metabolism, lignin biosynthesis, cellulose synthase activity, and other molecular functions. Additionally, WGCNA identified five co-expression modules significantly correlated with the main stem height, whose core genes encoded caffeoyl-CoA-O-methyltransferase, transcription factors ATAF2, WAT1, and GDSL lipase. In the present study, three modules (turquoise, blue and brown modules) had the highest correlation with cold tolerance phenotype and were selected for further analysis. The brown module had 108 hub genes, while the blue and turquoise models had 96 and 21 hub genes, respectively. Only 11 hub genes in the brown module had significantly higher expression levels under low-temperature stress, while those in the other two modules were significantly lower than those under control conditions. Through GO enrichment analysis, the results showed that hub genes were significantly enriched in biological pathways, such as sucrose synthase activity, translation initiation factor activity, UDP-glucosyltransferase activity, and UDP-glycosyltransferase activity, among which, the above biological pathways were all related to sucrose synthesis Enzymes and glycosyltransferases are related, thus indicating that the enzyme activity is involved in the low temperature response process and plays a crucial role. Through KEGG enrichment analysis, the results show that hub genes are mainly enriched in biological pathways such as Sphingolipid metabolism, Selenocompound metabolism, Sphingolipid metabolism, Starch and sucrose metabolism, Pentose phosphate pathway, Fructose and mannose metabolism, Galactose metabolism, Glycolysis, and Gluconeogenesis. Among them, the above biological pathways are all related to sugar metabolism, which indicates that monosaccharide or polysaccharide metabolism is involved in the process of *H. virescens* in response to low temperature stress. Therefore, the 11 hub genes screened in the brown module could be the key cold-tolerance genes of *H. virescens*, and might need further studies.

### The antioxidant defense system plays an important role in response to low-temperature stress in *Helictotrichon virescens*

Plants have complex mechanisms to respond to abiotic stresses (such as drought, salinity, and extreme temperatures). The stress signals are sensed by receptors on the cell membrane and then transduced to second messengers, a phenomenon that induces the activation of downstream physiological responses. The expression of different stress-responsive genes ultimately leads to a protective response in the whole plant (Varshney et al., 2011; Lei et al., 2014). Transcriptome profiling is an important strategy for elucidating the molecular components of cells and tissues for explaining functional elements of the genomes in response to different stimuli (Qiu et al., 2013). High-throughput RNA sequencing (RNA-seq) has been used for gene discovery and regulatory network studies, including stress response studies in higher plants (Deyholos, 2010; Kakumanu et al., 2012; Wang et al., 2013). In recent years, some studies have revealed the molecular mechanism of abiotic stress by RNA-seq technology in turfgrass. For example, comparative transcriptome analysis of tall fescue and ryegrass exposed to heat or cold stress for 10 h showed that approximately 30 and 25% of genes showed significant changes under heat and cold stress, respectively, and of them, *HSFs* genes exhibited strong responses to heat and cold stresses (Wang et al., 2015). Transcriptome data of cold-acclimated and non-acclimated bermudagrass were studied, and the results showed that 5,867 genes were differentially expressed in cold-acclimated and unaccustomed bermudagrass, of which 2,181 were downregulated and 710 were up-regulated; among up-regulated genes, *AP2*, *NAC,* and *WRKY* family members were associated with cold stress (Zhu et al., 2015). However, in comparison to other grasses, little research has been done on the molecular mechanism of *H. virescens* response to low-temperature stress. It is well known that low-temperature stress can change the permeability of mesophyll cells in crop seedlings, and the change in permeability is inversely proportional to the external temperature and proportional to the action time; therefore, the low-temperature stress seriously causes cell membrane damage and greatly changes cell membrane permeability (Fridovich, 1978) collectively referred to the three enzymes SOD, CAT, and POD as cytoprotective enzyme systems, and their activity trends could reflect the cold tolerance of crops. In this study, *H. virescens* seedlings were subjected to low temperature stress at 0°C, the results showed that after 12, 36, and 60 h of stress, the leaves of *H. virescens* seedlings suffered less low temperature damage and the plants could grow normally. Subsequently, this study conducted a method comparison and analysis of the phenotypic indicators of *H. virescens* under treated group and control group, the results showed that POD, SOD, CAT, and ROS were significantly different between the treatment and the control groups (*p* < 0.01; Table 3). The above results show that under low-temperature stress, the high activity of antioxidant enzymes is beneficial to the removal of ROS in *H. virescens* plants and reduces the damage of ROS in the plants.

In order to elucidate the mechanism of response to cold stress of *H. virescens*, previous research results show that: at low -temperature, ROS content accumulation was the same as POD, SOD and CAT activity, the gene that regulates POD activity (Cluster37118.16911) and the gene that regulates ROS (Cluster37118.62042) may be involved in ROS elimination during low-temperature treatment (Cheng et al., 2022). However, in this study, we used weighted gene co-expression network analysis (WGCNA) to identify the hub genes associated with cold tolerance in *H. virescens*. Specifically, the correlation coefficients between the brow module and superoxide dismutase, peroxisome, catalase, and ROS were 0.94 (*p* < 0.001), 0.89 (*p* < 0.001), 0.93 (*p* < 0.001), and 0.9 (<0.001; Figure 2).Therefore, GO annotation results show that, as shown in Figure 4A, the hub genes of cluster-37118.47362, cluster-37118.47713, and cluster-37118.66740 were mainly enriched in sucrose synthase, translation initiation factor, and biological pathways activities, such as UDP-glucosyltransferase, nitric-oxide synthase, glucosyltransferase, translation factor, structural molecule activities, and structural constituent of ribosome. KEGG annotation results show that, as shown in Figure 6A and Supplementary Table 4, the cluster-37118.47362, cluster-37118.47713, and cluster-37118.66740 were mainly enriched in sphingolipid, selenocompound, sphingolipid, starch, sucrose, pentose phosphate, fructose, mannose, and galactose metabolism pathways, and other biological pathways. Notably, these biological pathways are all related to glucose metabolism, showing that monosaccharide or polysaccharide metabolism is involved in *H. virescens* response to low-temperature stress. Subsequently, RT-PCR analysis of the hub genes that were highly expressed in the brown module showed that the expression levels of cluster-37118.47362, cluster-37118.47713, and cluster-37118.66740 were significantly higher than those in the control conditions at 36 h and 60 h of low-temperature treatments (Figure 7). Compared with the results of Cheng et al. (2022), in this study, the phenotypic data of the analysis results of POD, SOD, CAT, and ROS phenotype indicators were combined with the FPKM value of differential genes to conduct WGCNA analysis, and the key hub genes were mined. This analysis method is more reasonable and more purposeful, and at the same time, according to the analysis results of hub gene expression, GO annotation results, KEGG annotation results, it can showed that cluster-37118.47362, cluster-37118.47713, and cluster-37118.66740 play an important role in the response to low-temperature stress in the *H. virescens*. On the basis of the above research, bioinformatics analysis of hub gene showed that, the protein isoelectric points of the hubs were hydrophobic proteins, and the hub genes were all localized to the plasma membrane. By constructing a protein interaction network, it was found that the hub gene Cluster-37118.66740 interacted with Sb09g003460.1 and Sb04g020180.1 proteins in *Sorghum bicolor.* Homologous gene identification results showed that, a total of five sucrose synthase activity (SUS) family members were identified in *Sorghum bicolor*, six in *Oryza sativa Japonica*, and six *in Arabidopsis thaliana*. In addition, KEGG enrichment analysis were performed on the above homologous genes (Supplementary Tables 9–11), the results showed that, all homologous genes were significantly enriched in metabolic pathways and starch and sucrose metabolism pathways, and studies have shown that the above biological pathways are related to abiotic stress. It is worth noting that, by constructing phylogenetic trees of the four species of *H. virescens*, *Sorghum bicolo*, *Oryza sativa Japonica*, and *Arabidopsis thaliana*, the results showed that the gene Cluster 37118.66740 and Os03g0340500 belonged to the same ancestral branch and were in the same subfamily (Figure 9B), subsequently, we performed functional annotation of Os03g0340500, the functional annotation as Sucrose-UDP glucosyltransferase 4. In plants, UDP-glycosyltransferase catalyzes the glycosyl transfer reaction, which transfers the sugar group from the activated donor molecule to the acceptor molecule, thereby regulating the activity of the acceptor molecule in cells and organisms, such as biological activity, dissolution sex, and transport. In conclusion, the hub gene Cluster 37118.66740, as a key candidate gene, should be further analyzed in the follow-up studies.

## Data availability statement

The datasets presented in this study can be found in online repositories. The names of the repository/repositories and accession number(s) can be found at: https://www.ncbi.nlm.nih.gov/search/all/?term=PRJNA810780.

## Author contributions

MC, HW, RZ, XLei, MI, TY, XLi, YZ, YL, and RH designed the experiment. ZP and KC collated the transcriptome data. JZ and YC conducted the RT-PCR experiment. MC, XLei, TY, XLi, YZ, and YL drafted the manuscript. RH revised the manuscript. All authors contributed to the article and approved the submitted version.

## Funding

This study was supported by the Fundamental Research Funds for the Central Universities (ZYN2022053), Science and Technology Project of Sichuan Province (2020YJ0466), the Forage Innovation Team of Sichuan Province, and the Public Relations Project of Forage Breeding of Sichuan Province.

## Conflict of interest

The authors declare that the research was conducted in the absence of any commercial or financial relationships that could be construed as a potential conflict of interest.

## Publisher’s note

All claims expressed in this article are solely those of the authors and do not necessarily represent those of their affiliated organizations, or those of the publisher, the editors and the reviewers. Any product that may be evaluated in this article, or claim that may be made by its manufacturer, is not guaranteed or endorsed by the publisher.

## Footnotes

^1^ http://www.ncbi.nlm.nih.gov/dbEST/dbEST_summary.html

^2^ https://myhits.sib.swiss/cgi-bin/estscan

^3^ https://web.expasy.org/protparam/

^4^ https://wolfpsort.hgc.jp/

^5^ https://services.healthtech.dtu.dk/service.php?TMHMM-2.0

^6^ https://cn.string-db.org/
