# Supplementary material for: RNA sequencing and weighted gene co-expression network analysis uncover the hub genes controlling cold tolerance in Helictotrichon virescens seedlings
Source: Front Plant Sci. 2022 Sep 2;13:938859. doi: 10.3389/fpls.2022.938859 (PMC9478469; doi:10.3389/fpls.2022.938859)
Supplement: Supplementary file 1 [file Table_1.DOCX]

Supplementary paper 1 R package information used in the construction of weighted gene co-expression network

| **Name** | **Description** | **Version** |
| --- | --- | --- |
| BiocManager | Access the Bioconductor Project Package Repository | 1.30.16 |
| AnnotationDbi | Manipulation of SQLite-based annotations in Bioconductor | 1.56.2 |
| impute | impute: Imputation for microarray data | 1.68.0 |
| GO.db | A set of annotation maps describing the entire Gene Ontology | 3.14.0 |
| preprocessCore | A collection of pre-processing functions | 1.56.0 |
| WGCNA | Weighted Correlation Network Analysis | 1.70.3 |
