# Supplementary material for: RNA sequencing and weighted gene co-expression network analysis uncover the hub genes controlling cold tolerance in Helictotrichon virescens seedlings
Source: Front Plant Sci. 2022 Sep 2;13:938859. doi: 10.3389/fpls.2022.938859 (PMC9478469; doi:10.3389/fpls.2022.938859)
Supplement: Supplementary file 2 [file Table_2.DOCX]

| Supplementary Paper 2 Components of reaction | | | |
| --- | --- | --- | --- |
| Remove genomic contamination system in RNA | | Reverse transcription system | |
| Reagent | Volume (μL) | Reagent | Volume (μL) |
| 10X Reaction Buffer | 1 | 5X Reaction Buffer | 4 |
| with MgCl_2_ | 1 | Oligo (dT) 18 primer | 1 |
| total RNA | 1 | 10 mM dNTP Mix | 2 |
| DNase I | 1 | total RNA | 1 |
| Water, nuclease-free | 7 | RiboLock Rnase Inhibitor | 1 |
|  |  | RevertAid M-MuLV RT | 1 |
|  |  | Water, nuclease-free | 10 |
| Total | 10 | Total | 20 |
