# Supplementary material for: RNA sequencing and weighted gene co-expression network analysis uncover the hub genes controlling cold tolerance in Helictotrichon virescens seedlings
Source: Front Plant Sci. 2022 Sep 2;13:938859. doi: 10.3389/fpls.2022.938859 (PMC9478469; doi:10.3389/fpls.2022.938859)
Supplement: Supplementary file 3 [file Table_3.DOCX]

| Supplementary Paper3 Components of qRT-PCR reaction | |
| --- | --- |
| Reagent | Volume (μL) |
| ddH_2_O_2_ | 5 |
| 2X SYBR Green Fast qPCR Mix | 7.5 |
| Forward primer（10μM） | 0.4 |
| Reverse primer（10μM） | 0.4 |
| cDNA | 1 |
| Total | 10 |
