# Supplementary material for: RNA sequencing and weighted gene co-expression network analysis uncover the hub genes controlling cold tolerance in Helictotrichon virescens seedlings
Source: Front Plant Sci. 2022 Sep 2;13:938859. doi: 10.3389/fpls.2022.938859 (PMC9478469; doi:10.3389/fpls.2022.938859)
Supplement: Supplementary file 4 [file Table_4.DOCX]

| Supplementary Paper 4 Reaction Step | | |
| --- | --- | --- |
| Step | Temperature | Time |
| 1 | 95 ℃ | 3 min |
| 2 | 95 ℃ | 5s |
| 3 | 60 ℃ | 30 s |
| 4 | ﹢H10Plate Read，Go to step 2 | 40-45 cycles |
| 5 | Melt Curve 65-95℃ |  |
| 6 | ﹢Plate Read，increment 0.5℃ | 0.5 s |
| 7 | end |  |
