# Supplementary material for: RNA sequencing and weighted gene co-expression network analysis uncover the hub genes controlling cold tolerance in Helictotrichon virescens seedlings
Source: Front Plant Sci. 2022 Sep 2;13:938859. doi: 10.3389/fpls.2022.938859 (PMC9478469; doi:10.3389/fpls.2022.938859)
Supplement: Supplementary file 5 [file Table_5.DOCX]

| Supplementary Paper 5 Primers used in RT-PCR | | |
| --- | --- | --- |
| Gene | Forward primer | Reverse primer |
| Cluster-37118.47362 | CCCACTTTCCCTAACCCATTAC | CGGACTATCCTTCTGAACAACC |
| Cluster-37118.47713 | GGAGCATCTCCAGGTCAAATAC | GCCTCGATGTAATCCTGAATGT |
| Cluster-37118.66740 | GACCTGTGGACTTCCTACTTTC | CAGGATGGTAAGGATCGATGTG |
| Cluster-37118.28125 | GAAGTACAGGATCAGGCTAAGTG | GTCGTAGACATGAACCGTCAG |
