# Supplementary material for: RNA sequencing and weighted gene co-expression network analysis uncover the hub genes controlling cold tolerance in Helictotrichon virescens seedlings
Source: Front Plant Sci. 2022 Sep 2;13:938859. doi: 10.3389/fpls.2022.938859 (PMC9478469; doi:10.3389/fpls.2022.938859)
Supplement: Supplementary file 6 [file Table_6.DOCX]

Supplement Table 1 GO enrichment analysis of hub gene in brown module (MF of Top 20)

| Description | pValue | Gene_names |
| --- | --- | --- |
| sucrose synthase activity | 0.0001292 | Cluster-37118.66740,Cluster-37118.7361 |
| translation initiation factor activity | 0.0017086 | Cluster-37118.42165,Cluster-37118.46066 |
| UDP-glucosyltransferase activity | 0.0024638 | Cluster-37118.66740,Cluster-37118.7361 |
| nitric-oxide synthase activity | 0.0029419 | Cluster-37118.37348 |
| glucosyltransferase activity | 0.0031829 | Cluster-37118.66740,Cluster-37118.7361 |
| translation factor activity, RNA binding | 0.0048914 | Cluster-37118.42165,Cluster-37118.46066 |
| UDP-glycosyltransferase activity | 0.0085254 | Cluster-37118.66740,Cluster-37118.7361 |
| protein-arginine deiminase activity | 0.0085668 | Cluster-37118.18433 |
| NAD+ kinase activity | 0.0095967 | Cluster-37118.47362 |
| actin filament binding | 0.011342 | Cluster-37118.48621 |
| hydrolase activity | 0.012505 | Cluster-37118.18433 |
| voltage-gated chloride channel activity | 0.01374 | Cluster-37118.48621 |
| chloride channel activity | 0.01374 | Cluster-37118.48621 |
| voltage-gated anion channel activity | 0.01374 | Cluster-37118.48621 |
| chloride transmembrane transporter activity | 0.01374 | Cluster-37118.48621 |
| protein binding, bridging | 0.014315 | Cluster-37118.48621 |
| 6-phosphofructokinase activity | 0.015373 | Cluster-37118.47362 |
| anion channel activity | 0.017687 | Cluster-37118.48621 |
| structural molecule activity | 0.019361 | Cluster-37118.46066,Cluster-37118.14533,Cluster-37118.14130,Cluster-37118.28125 |
| structural constituent of ribosome | 0.019508 | Cluster-37118.14130,Cluster-37118.46066,Cluster-37118.14533 |
