# Supplementary material for: RNA sequencing and weighted gene co-expression network analysis uncover the hub genes controlling cold tolerance in Helictotrichon virescens seedlings
Source: Front Plant Sci. 2022 Sep 2;13:938859. doi: 10.3389/fpls.2022.938859 (PMC9478469; doi:10.3389/fpls.2022.938859)
Supplement: Supplementary file 7 [file Table_7.DOCX]

Supplement Table 2 GO enrichment analysis of hub gene in blue module (MF of Top 20)

| Description | pValue | Gene_names |
| --- | --- | --- |
| glycine reductase activity | 0.0016489 | Cluster-37118.46642 |
| proline-tRNA ligase activity | 0.0046401 | Cluster-37118.47341 |
| oxidoreductase activity | 0.0055874 | Cluster-37118.46642 |
| oxidoreductase activity | 0.0055874 | Cluster-37118.46642 |
| sodium:proton antiporter activity | 0.0070533 | Cluster-37118.45930 |
| nucleotide-sugar transmembrane transporter activity | 0.0077008 | Cluster-37118.46409 |
| pyrimidine nucleotide-sugar transmembrane transporter activity | 0.0077008 | Cluster-37118.46409 |
| asparagine synthase (glutamine-hydrolyzing) activity | 0.0086209 | Cluster-37118.52628 |
| monovalent cation:proton antiporter activity | 0.0091915 | Cluster-37118.45930 |
| cation:cation antiporter activity | 0.0091915 | Cluster-37118.45930 |
| phosphatase activity | 0.010679 | Cluster-37118.52427,Cluster-37118.42974 |
| nucleotide transmembrane transporter activity | 0.010928 | Cluster-37118.46409 |
| organophosphate ester transmembrane transporter activity | 0.010928 | Cluster-37118.46409 |
| acid phosphatase activity | 0.012679 | Cluster-37118.52427 |
| molybdate ion transmembrane transporter activity | 0.015298 | Cluster-37118.41399 |
| carbon-nitrogen ligase activity, with glutamine as amido-N-donor | 0.015533 | Cluster-37118.52628 |
| phosphate transmembrane transporter activity | 0.017388 | Cluster-37118.46409 |
| phosphoric ester hydrolase activity | 0.017751 | Cluster-37118.42974,Cluster-37118.52427 |
| carbohydrate derivative transporter activity | 0.021479 | Cluster-37118.46409 |
| solute:cation antiporter activity | 0.026119 | Cluster-37118.45930 |
