# Supplementary material for: RNA sequencing and weighted gene co-expression network analysis uncover the hub genes controlling cold tolerance in Helictotrichon virescens seedlings
Source: Front Plant Sci. 2022 Sep 2;13:938859. doi: 10.3389/fpls.2022.938859 (PMC9478469; doi:10.3389/fpls.2022.938859)
Supplement: Supplementary file 8 [file Table_8.DOCX]

Supplement Table 3 GO enrichment analysis of hub gene in turquoise module (MF of Top 20)

| Description | pValue | Gene_names |
| --- | --- | --- |
| glucose-6-phosphate dehydrogenase activity | 0.003232 | Cluster-37118.55857 |
| peptide-methionine (R)-S-oxide reductase activity | 0.006544 | Cluster-37118.46249 |
| glutamate synthase activity | 0.006868 | Cluster-37118.46249 |
| nitronate monooxygenase activity | 0.007985 | Cluster-37118.46249 |
| oxidoreductase activity | 0.008281 | Cluster-37118.55857,Cluster-37118.45675 |
| dephospho-CoA kinase activity | 0.008496 | Cluster-37118.38354 |
| oxidoreductase activity, acting on CH-OH group of donors | 0.009879 | Cluster-37118.55857,Cluster-37118.45675 |
| oxidoreductase activity, acting on a sulfur group of donors | 0.01039 | Cluster-37118.46249 |
| monosaccharide binding | 0.013646 | Cluster-37118.47615 |
| oxidoreductase activityfunction oxidases) | 0.013939 | Cluster-37118.46249 |
| oxidoreductase activity | 0.024826 | Cluster-37118.55857,Cluster-37118.45675,Cluster-37118.45412,Cluster-37118.46249 |
| oxidoreductase activity, acting on the CH-NH2 group of donors | 0.026133 | Cluster-37118.46249 |
| nutrient reservoir activity | 0.027203 | Cluster-37118.46437 |
| 3-hydroxyacyl-CoA dehydrogenase activity | 0.033454 | Cluster-37118.45675 |
| oxidoreductase activity, acting on a sulfur group of donors | 0.049982 | Cluster-37118.46249 |
| NADP binding | 0.051445 | Cluster-37118.55857 |
| oxidoreductase activity | 0.058498 | Cluster-37118.46249 |
| monooxygenase activity | 0.066045 | Cluster-37118.46249 |
