# Supplementary material for: RNA sequencing and weighted gene co-expression network analysis uncover the hub genes controlling cold tolerance in Helictotrichon virescens seedlings
Source: Front Plant Sci. 2022 Sep 2;13:938859. doi: 10.3389/fpls.2022.938859 (PMC9478469; doi:10.3389/fpls.2022.938859)
Supplement: Supplementary file 9 [file Table_9.DOCX]

Supplement Table 4 KEGG enrichment analysis of hub gene in brown module

| Term | ID | P-Value | Input |
| --- | --- | --- | --- |
| Other glycan degradation | ko00511 | 0.0528625 | Cluster-37118.58013 |
| Selenocompound metabolism | ko00450 | 0.0649685 | Cluster-37118.28588 |
| Sphingolipid metabolism | ko00600 | 0.0658937 | Cluster-37118.58013 |
| Starch and sucrose metabolism | ko00500 | 0.091697 | Cluster-37118.47713\|Cluster-37118.66740 |
| Basal transcription factors | ko03022 | 0.1057633 | Cluster-37118.67167 |
| Pentose phosphate pathway | ko00030 | 0.1285668 | Cluster-37118.47362 |
| Fructose and mannose metabolism | ko00051 | 0.1363325 | Cluster-37118.47362 |
| Galactose metabolism | ko00052 | 0.1448849 | Cluster-37118.47362 |
| Cyanoamino acid metabolism | ko00460 | 0.1953594 | Cluster-37118.47713 |
| RNA degradation | ko03018 | 0.2112909 | Cluster-37118.47362 |
| Pyrimidine metabolism | ko00240 | 0.2238227 | Cluster-37118.28588 |
| Glyoxylate and dicarboxylate metabolism | ko00630 | 0.2415104 | Cluster-37118.56946 |
| Glycolysis / Gluconeogenesis | ko00010 | 0.300839 | Cluster-37118.47362 |
| RNA transport | ko03013 | 0.3127252 | Cluster-37118.42165 |
| Phenylpropanoid biosynthesis | ko00940 | 0.3629384 | Cluster-37118.47713 |
