# Supplementary material for: RNA sequencing and weighted gene co-expression network analysis uncover the hub genes controlling cold tolerance in Helictotrichon virescens seedlings
Source: Front Plant Sci. 2022 Sep 2;13:938859. doi: 10.3389/fpls.2022.938859 (PMC9478469; doi:10.3389/fpls.2022.938859)
Supplement: Supplementary file 10 [file Table_10.DOCX]

Supplement Table 5 KEGG enrichment analysis of hub gene in blue module

| Term | ID | P-Value | Input |
| --- | --- | --- | --- |
| Endocytosis | ko04144 | 0.0076694 | Cluster-37118.46093\|Cluster-37118.51138 |
