# Supplementary material for: RNA sequencing and weighted gene co-expression network analysis uncover the hub genes controlling cold tolerance in Helictotrichon virescens seedlings
Source: Front Plant Sci. 2022 Sep 2;13:938859. doi: 10.3389/fpls.2022.938859 (PMC9478469; doi:10.3389/fpls.2022.938859)
Supplement: Supplementary file 11 [file Table_11.DOCX]

Supplement Table 6 KEGG enrichment analysis of hub gene in turquoise module

| Term | ID | P-Value | Input |
| --- | --- | --- | --- |
| Carotenoid biosynthesis | ko00906 | 0.002658 | Cluster-37118.46100\|Cluster-37118.45512 |
| Glyoxylate and dicarboxylate metabolism | ko00630 | 0.026657 | Cluster-37118.46249\|Cluster-37118.45675 |
