# Supplementary material for: RNA sequencing and weighted gene co-expression network analysis uncover the hub genes controlling cold tolerance in Helictotrichon virescens seedlings
Source: Front Plant Sci. 2022 Sep 2;13:938859. doi: 10.3389/fpls.2022.938859 (PMC9478469; doi:10.3389/fpls.2022.938859)
Supplement: Supplementary file 12 [file Table_12.DOCX]

Supplementary Table 7 Homologous gene identification of key gene expression sequences

| Query ID | Scientific Name | Query Cover | Ident | Accession |
| --- | --- | --- | --- | --- |
| Cluster-37118.28125 | Avena barbata | 31% | 96.94% | GR340692.1 |
|  | Triticum aestivum | 49% | 86.30% | CJ688921.1 |
|  | Triticum aestivum | 49% | 86.12% | CD939628.1 |
|  | Lolium perenne | 34% | 92.43% | JZ165798.1 |
|  | Triticum aestivum | 39% | 90.74% | HX069915.1 |
|  | Hordeum vulgare subsp. vulgare | 35% | 90.46% | GH227840.1 |
|  | Triticum aestivum | 35% | 90.12% | CJ898754.1 |
|  | Triticum aestivum | 35% | 90.12% | CJ898445.1 |
|  | Hordeum vulgare subsp. vulgare | 45% | 85.51% | BM372437.2 |
|  | Leymus cinereus x Leymus triticoides | 32% | 91.67% | EG384316.1 |
|  | Triticum aestivum | 34% | 90.00% | LU040853.1 |
|  | Hordeum vulgare | 34% | 89.16% | BE412718.1 |
|  | Hordeum vulgare subsp. vulgare | 38% | 86.72% | DK710387.1 |
|  | Hordeum vulgare subsp. vulgare | 38% | 86.72% | CB883534.1 |
|  | Hordeum vulgare subsp. vulgare | 36% | 87.10% | DK620133.1 |
|  | Hordeum vulgare subsp. vulgare | 32% | 89.47% | AL503566.1 |
|  | Leymus cinereus x Leymus triticoides | 36% | 86.59% | EG401042.1 |
|  | Leymus cinereus x Leymus triticoides | 36% | 86.59% | EG392358.1 |
|  | Brachypodium distachyon | 34% | 87.43% | HX805757.1 |
|  | Triticum aestivum | 30% | 89.75% | CJ582466.1 |
|  | Hordeum vulgare subsp. vulgare | 31% | 87.07% | DK637882.1 |
| Cluster-37118.66740 | Lolium multiflorum | 79% | 96.31% | AU246862.1 |
|  | Triticum aestivum | 84% | 93.97% | HX045248.1 |
|  | Triticum aestivum | 84% | 93.53% | LU102136.1 |
|  | Triticum aestivum | 84% | 93.53% | LU102758.1 |
|  | Triticum aestivum | 84% | 93.53% | LU102726.1 |
|  | Triticum aestivum | 84% | 93.53% | LU102812.1 |
|  | Triticum aestivum | 84% | 93.53% | LU094189.1 |
|  | Triticum aestivum | 84% | 93.53% | CJ700348.1 |
|  | Triticum aestivum | 84% | 93.53% | CJ705711.1 |
|  | Triticum aestivum | 84% | 93.53% | BJ259821.1 |
|  | Triticum aestivum | 84% | 93.51% | CJ698508.1 |
|  | Hordeum vulgare subsp. vulgare | 84% | 93.48% | DK742930.1 |
|  | Hordeum vulgare subsp. vulgare | 84% | 93.48% | DK697400.1 |
|  | Hordeum vulgare subsp. vulgare | 84% | 93.48% | DK600269.1 |
|  | Hordeum vulgare subsp. vulgare | 84% | 93.48% | CA004691.1 |
|  | Triticum aestivum | 84% | 92.67% | CJ717015.1 |
|  | Triticum aestivum | 84% | 92.67% | CJ719625.1 |
|  | Triticum aestivum | 81% | 93.72% | CJ716840.1 |
|  | Triticum aestivum | 84% | 90.64% | CJ719957.1 |
|  | Triticum aestivum | 84% | 90.52% | CA729700.1 |
|  | Oryza sativa Indica Group | 83% | 89.52% | CT863502.1 |
|  | Oryza sativa Japonica Group | 83% | 89.52% | CI752100.1 |
|  | Oryza sativa Japonica Group | 83% | 89.52% | CI608008.1 |
|  | Oryza sativa Japonica Group | 83% | 89.52% | CI303728.1 |
|  | Oryza sativa Japonica Group | 83% | 89.52% | CB648734.1 |
|  | Oryza sativa Japonica Group | 83% | 89.52% | CB645387.1 |
|  | Oryza sativa Japonica Group | 83% | 89.52% | CB639729.1 |
|  | Oryza sativa Indica Group | 83% | 89.52% | CB635134.1 |
|  | Oryza sativa Indica Group | 83% | 89.52% | CB631361.1 |
|  | Oryza sativa Indica Group | 83% | 89.52% | CB631302.1 |
|  | Oryza sativa Indica Group | 83% | 89.52% | CB628938.1 |
|  | Oryza sativa Indica Group | 83% | 89.52% | CB627276.1 |
|  | Oryza sativa/Pyricularia oryzae mixed EST library | 83% | 89.52% | AW155001.1 |
|  | Panicum virgatum | 84% | 89.18% | FL805130.1 |
|  | Panicum virgatum | 84% | 89.18% | FL802595.1 |
|  | Panicum virgatum | 84% | 89.18% | FL697088.1 |
|  | Oryza sativa Japonica Group | 79% | 90.32% | CI763820.1 |
|  | Oryza sativa Japonica Group | 83% | 89.08% | CI755186.1 |
|  | Oryza sativa Japonica Group | 83% | 89.08% | CI602672.1 |
|  | Oryza sativa Japonica Group | 83% | 89.08% | CI609047.1 |
|  | Oryza sativa Japonica Group | 83% | 89.08% | CB641138.1 |
|  | Oryza sativa Japonica Group | 83% | 88.65% | CI745864.1 |
|  | Oryza sativa Japonica Group | 83% | 88.65% | CI760257.1 |
|  | Panicum virgatum | 84% | 88.31% | FL862051.1 |
|  | Panicum virgatum | 84% | 88.31% | FL819332.1 |
|  | Oryza sativa Japonica Group | 76% | 90.00% | CI585914.1 |
|  | Oryza sativa Japonica Group | 83% | 87.77% | CI621426.1 |
|  | Sorghum propinquum | 84% | 87.01% | BF481989.1 |
|  | Oryza sativa Japonica Group | 83% | 86.03% | CI741028.1 |
|  | Oryza sativa Japonica Group | 64% | 91.53% | CB640603.1 |
|  | Oryza sativa Indica Group | 56% | 90.91% | CB633383.1 |
|  | Oryza sativa Japonica Group | 53% | 87.76% | CI616680.1 |
| Cluster-37118.47713 | Avena barbata | 32% | 94.87% | GR351386.1 |
|  | Avena barbata | 32% | 94.87% | GR352686.1 |
|  | Avena barbata | 32% | 94.87% | GR345775.1 |
|  | Avena barbata | 31% | 93.42% | GR349698.1 |
|  | Avena barbata | 28% | 94.29% | GR343797.1 |
| Cluster-37118.47362 | Triticum aestivum | 73% | 89.72% | HX132330.1 |
|  | Triticum aestivum | 67% | 89.10% | HX144193.1 |
|  | Triticum aestivum | 62% | 90.57% | CJ687394.1 |
|  | Triticum aestivum | 62% | 90.26% | CV762808.1 |
|  | Hordeum vulgare subsp. vulgare | 76% | 85.76% | BI953706.1 |
|  | Panicum virgatum | 68% | 86.90% | FL979462.1 |
|  | Triticum aestivum | 56% | 91.13% | CJ730159.1 |
|  | Triticum aestivum | 51% | 92.70% | CK152782.1 |
|  | Triticum aestivum | 51% | 92.70% | CK151483.1 |
|  | Triticum aestivum | 53% | 91.62% | BJ246660.1 |
|  | Hordeum vulgare subsp. vulgare | 52% | 92.28% | CA007070.1 |
|  | Zea mays | 68% | 86.07% | DR814757.1 |
|  | Triticum aestivum | 51% | 92.35% | CK152501.1 |
|  | Triticum aestivum | 49% | 93.27% | JZ891628.1 |
|  | Triticum aestivum | 59% | 88.08% | HX189137.1 |
|  | Lolium arundinaceum | 50% | 91.92% | DT695274.1 |
|  | Oryza sativa Japonica Group | 62% | 87.14% | CB681147.1 |
|  | Triticum aestivum | 52% | 90.02% | CJ647319.1 |
|  | Zea mays | 60% | 87.21% | DY398346.1 |
|  | Hordeum vulgare subsp. vulgare | 49% | 91.59% | BQ465299.1 |
|  | Brachypodium distachyon | 46% | 93.13% | GT841327.1 |
|  | Saccharum hybrid cultivar SP80-3280 | 56% | 88.61% | CA141150.1 |
|  | Pseudoroegneria spicata | 58% | 87.78% | FF350673.1 |
|  | Panicum virgatum | 61% | 86.82% | JG905055.1 |
|  | Panicum virgatum | 56% | 88.54% | FL980988.1 |
|  | Triticum aestivum | 45% | 93.54% | BQ294662.1 |
|  | Hordeum vulgare subsp. vulgare | 49% | 91.20% | AW982559.3 |
|  | Brachypodium distachyon | 45% | 93.33% | GT787380.1 |
|  | Setaria italica | 53% | 89.15% | JK551726.1 |
|  | Hordeum vulgare subsp. vulgare | 59% | 87.37% | BG367393.1 |
|  | Triticum aestivum | 51% | 89.85% | LU060557.1 |
|  | Brachypodium distachyon | 54% | 88.46% | DV486799.1 |
|  | Triticum aestivum | 44% | 93.04% | HX200678.1 |
|  | Zea mays | 55% | 87.86% | FM190236.1 |
|  | Saccharum hybrid cultivar SP80-3280 | 57% | 87.23% | CA208337.1 |
|  | Triticum aestivum | 55% | 87.86% | CJ645659.1 |
|  | Lolium perenne | 49% | 90.50% | GR520055.1 |
|  | Zea mays | 59% | 86.47% | BM952618.1 |
|  | Saccharum hybrid cultivar (mixed) | 59% | 86.27% | CA227378.1 |
|  | Avena barbata | 54% | 88.47% | GR334529.1 |
|  | Triticum aestivum | 43% | 93.20% | CD913159.1 |
|  | Saccharum hybrid cultivar CoS 767 | 55% | 87.36% | DN195684.1 |
|  | Triticum aestivum | 43% | 92.98% | AJ604444.1 |
|  | Saccharum hybrid cultivar (mixed) | 49% | 89.46% | CA279677.1 |
|  | Triticum aestivum | 42% | 93.04% | AL813910.1 |
|  | Hordeum vulgare subsp. vulgare | 43% | 91.75% | AL504756.1 |
|  | Hordeum vulgare subsp. vulgare | 52% | 87.95% | BF261099.2 |
|  | Festuca pratensis | 52% | 87.14% | GO891614.1 |
|  | Saccharum hybrid cultivar (mixed) | 49% | 88.45% | CA299447.1 |
|  | Triticum aestivum | 54% | 86.50% | HX176721.1 |
|  | Lolium arundinaceum | 48% | 88.89% | DT688919.1 |
|  | Brachypodium distachyon | 39% | 93.50% | DV474684.1 |
|  | Triticum aestivum | 54% | 86.54% | HX143324.1 |
|  | Saccharum hybrid cultivar SP80-3280 | 50% | 87.35% | CA157062.1 |
|  | Lolium arundinaceum | 47% | 88.72% | DT689346.1 |
|  | Brachypodium distachyon | 39% | 93.36% | DV487629.1 |
|  | Panicum virgatum | 49% | 87.80% | FL890620.1 |
|  | Panicum virgatum | 45% | 89.24% | JG912396.1 |
|  | Saccharum hybrid cultivar (mixed) | 50% | 86.88% | CA300235.1 |
|  | Zea mays | 55% | 85.05% | EC895180.2 |
|  | Brachypodium distachyon | 49% | 87.43% | DV472047.1 |
|  | Panicum virgatum | 43% | 89.75% | HO291360.1 |
|  | Triticum aestivum | 52% | 86.05% | CJ728978.1 |
|  | Hordeum vulgare | 43% | 89.86% | AJ476473.1 |
|  | Triticum aestivum | 47% | 87.55% | LU085696.1 |
|  | Saccharum hybrid cultivar SP80-3280 | 53% | 85.26% | CA073357.1 |
|  | Panicum virgatum | 43% | 89.00% | JG921805.1 |
|  | Hordeum vulgare subsp. vulgare | 36% | 92.68% | BU972574.1 |
|  | Panicum virgatum | 40% | 90.22% | HO298389.1 |
|  | Panicum virgatum | 51% | 85.12% | JG860113.1 |
|  | Triticum aestivum | 45% | 87.28% | HX173081.1 |
|  | Hordeum vulgare subsp. vulgare | 38% | 91.08% | BU974462.1 |
|  | Triticum turgidum subsp. durum | 35% | 93.13% | BE428392.1 |
|  | Panicum virgatum | 44% | 86.97% | FL870792.1 |
|  | Saccharum hybrid cultivar SP80-3280 | 35% | 92.19% | CA175724.1 |
|  | Zea mays | 44% | 86.43% | CB329630.1 |
|  | Hordeum vulgare subsp. vulgare | 44% | 87.25% | BQ753389.1 |
|  | Hordeum vulgare subsp. vulgare | 44% | 87.22% | DK680898.1 |
|  | Hordeum vulgare subsp. vulgare | 44% | 87.22% | DK628269.1 |
|  | Panicum virgatum | 41% | 87.90% | JG965150.1 |
|  | Triticum aestivum | 40% | 87.96% | CA604742.1 |
|  | Zea mays | 42% | 87.29% | BE050732.1 |
|  | Panicum virgatum | 34% | 91.71% | FL979761.1 |
|  | Saccharum hybrid cultivar CoS 767 | 36% | 90.07% | CN607215.1 |
|  | Hordeum vulgare subsp. vulgare | 45% | 85.85% | CA006555.1 |
|  | Triticum aestivum | 31% | 93.22% | CJ663620.1 |
|  | Oryza sativa Japonica Group | 33% | 91.03% | D41203.1 |
|  | Oryza sativa Japonica Group | 35% | 90.13% | D40601.1 |
|  | Agrostis capillaris | 30% | 94.15% | DV854236.1 |
|  | Lolium perenne | 43% | 85.92% | GR524273.1 |
|  | Saccharum hybrid cultivar CoS 767 | 36% | 88.83% | DV731469.1 |
|  | Triticum aestivum | 41% | 86.15% | CJ710736.1 |
