# Supplementary material for: RNA sequencing and weighted gene co-expression network analysis uncover the hub genes controlling cold tolerance in Helictotrichon virescens seedlings
Source: Front Plant Sci. 2022 Sep 2;13:938859. doi: 10.3389/fpls.2022.938859 (PMC9478469; doi:10.3389/fpls.2022.938859)
Supplement: Supplementary file 13 [file Table_13.DOCX]

Supplementary Table 8. Hub gene family identification

| Subject id | Query id | % identity | alignment length | mismatches | gap openings | q. Start | q. End | s. Start | s. End | E value | score |
| --- | --- | --- | --- | --- | --- | --- | --- | --- | --- | --- | --- |
| Cluster-37118.66740 | SORBI_3001G378300 | 91.542 | 733 | 62 | 0 | 10 | 742 | 75 | 807 | 0 | 1428 |
| Cluster-37118.66740 | SORBI_3010G072300 | 72.951 | 732 | 197 | 1 | 10 | 741 | 72 | 802 | 0 | 1124 |
| Cluster-37118.66740 | SORBI_3001G344500 | 71.995 | 732 | 204 | 1 | 10 | 741 | 80 | 810 | 0 | 1120 |
| Cluster-37118.66740 | SORBI_3010G276700 | 60.846 | 733 | 280 | 4 | 10 | 737 | 120 | 850 | 0 | 938 |
| Cluster-37118.66740 | SORBI_3004G357600 | 56.486 | 740 | 307 | 4 | 11 | 737 | 74 | 811 | 0 | 832 |
| Cluster-37118.66740 | Os03g0340500 | 91.701 | 735 | 61 | 0 | 10 | 744 | 75 | 809 | 0 | 1435 |
| Cluster-37118.66740 | Os06g0194900 | 72.541 | 732 | 200 | 1 | 10 | 741 | 72 | 802 | 0 | 1132 |
| Cluster-37118.66740 | Os03g0401300 | 71.585 | 732 | 207 | 1 | 10 | 741 | 80 | 810 | 0 | 1118 |
| Cluster-37118.66740 | Os07g0616800 | 70.219 | 732 | 217 | 1 | 10 | 741 | 80 | 810 | 0 | 1116 |
| Cluster-37118.66740 | Os04g0249500 | 59.023 | 737 | 285 | 4 | 10 | 742 | 78 | 801 | 0 | 915 |
| Cluster-37118.66740 | Os04g0309600 | 58.752 | 737 | 287 | 4 | 10 | 742 | 78 | 801 | 0 | 911 |
| Cluster-37118.66740 | AT4G02280 | 80.874 | 732 | 140 | 0 | 11 | 742 | 78 | 809 | 0 | 1283 |
| Cluster-37118.66740 | AT5G49190 | 76.262 | 733 | 174 | 0 | 10 | 742 | 74 | 806 | 0 | 1212 |
| Cluster-37118.66740 | AT3G43190 | 70.941 | 733 | 212 | 1 | 10 | 742 | 77 | 808 | 0 | 1102 |
| Cluster-37118.66740 | AT5G20830 | 69.85 | 733 | 220 | 1 | 10 | 742 | 77 | 808 | 0 | 1069 |
| Cluster-37118.66740 | AT1G73370 | 58.992 | 734 | 300 | 1 | 10 | 742 | 80 | 813 | 0 | 922 |
| Cluster-37118.66740 | AT5G37180 | 57.859 | 738 | 307 | 2 | 5 | 741 | 67 | 801 | 0 | 906 |
