# Supplementary material for: RNA sequencing and weighted gene co-expression network analysis uncover the hub genes controlling cold tolerance in Helictotrichon virescens seedlings
Source: Front Plant Sci. 2022 Sep 2;13:938859. doi: 10.3389/fpls.2022.938859 (PMC9478469; doi:10.3389/fpls.2022.938859)
Supplement: Supplementary file 14 [file Table_14.DOCX]

Supplementary Table 9. Homologous gene KEGG enrichment analysis (*Sorghum bicolor*)

| Query: | Gene: | Pathway: | |
| --- | --- | --- | --- |
| SORBI_3001G344500 | sbi:8077078 | Metabolic pathways | Starch and sucrose metabolism |
| SORBI_3001G378300 | sbi:8082319 | Metabolic pathways | Starch and sucrose metabolism |
| SORBI_3004G357600 | sbi:8056263 | Metabolic pathways | Starch and sucrose metabolism |
| SORBI_3010G072300 | sbi:110431014 | Metabolic pathways | Starch and sucrose metabolism |
| SORBI_3010G276700 | sbi:110431016 | Metabolic pathways | Starch and sucrose metabolism |
