# Supplementary material for: RNA sequencing and weighted gene co-expression network analysis uncover the hub genes controlling cold tolerance in Helictotrichon virescens seedlings
Source: Front Plant Sci. 2022 Sep 2;13:938859. doi: 10.3389/fpls.2022.938859 (PMC9478469; doi:10.3389/fpls.2022.938859)
Supplement: Supplementary file 15 [file Table_15.DOCX]

Supplementary Table 10. Homologous gene KEGG enrichment analysis (*Arabidopsis thaliana*)

| Query: | Gene: | Pathway: | |
| --- | --- | --- | --- |
| AT1G73370 | ath:AT1G73370 | Metabolic pathways | Starch and sucrose metabolism |
| AT3G43190 | ath:AT3G43190 | Metabolic pathways | Starch and sucrose metabolism |
| AT4G02280 | ath:AT4G02280 | Metabolic pathways | Starch and sucrose metabolism |
| AT5G20830 | ath:AT5G20830 | Metabolic pathways | Starch and sucrose metabolism |
| AT5G37180 | ath:AT5G37180 | Metabolic pathways | Starch and sucrose metabolism |
| AT5G49190 | ath:AT5G49190 | Metabolic pathways | Starch and sucrose metabolism |

Supplementary Table 11. Homologous gene KEGG enrichment analysis (*Oryza sativa Japonica*)

| Query: | Gene: | Pathway: | |
| --- | --- | --- | --- |
| Os03g0340500 | osa:4332788 | Metabolic pathways | Starch and sucrose metabolism |
| Os03g0401300 | osa:4333062 | Metabolic pathways | Starch and sucrose metabolism |
| Os04g0249500 | osa:4335303 | Metabolic pathways | Starch and sucrose metabolism |
| Os04g0309600 | osa:4335447 | Metabolic pathways | Starch and sucrose metabolism |
| Os06g0194900 | osa:4340386 | Metabolic pathways | Starch and sucrose metabolism |
| Os07g0616800 | osa:4343910 | Metabolic pathways | Starch and sucrose metabolism |
